# A novel targeted co-delivery nanosystem for enhanced ovarian cancer treatment via multidrug resistance reversion and mTOR-mediated signaling pathway

**DOI:** 10.1186/s12951-021-01139-1

**Published:** 2021-12-23

**Authors:** Xueqin Wang, Tiandi Xiong, Miao Cui, Na Li, Qin Li, Li Zhu, Shaofeng Duan, Yunlong Wang, Yuqi Guo

**Affiliations:** 1 Henan Provincial People’s Hospital, Zhengzhou, 450003 China; 2 College of Bioengineering, Henan University of Technology, Zhengzhou, 450001 China; 3 People’s Hospital of Zhengzhou University, Zhengzhou, 450003 China; 4 Henan International Joint Laboratory for Gynecological Oncology and Nanomedicine, Zhengzhou, 450003 China; 5 Institute for Innovative Drug Design and Evaluation, School of Pharmacy, Henan University, Kaifeng, 475004 China; 6 Henan International Joint Laboratory of Chinese Medicine Efficacy, Henan University, Kaifeng, 475004 China; 7 Henan Bioengineering Research Center, Zhengzhou, 450046 China

**Keywords:** Ovarian cancer, Multidrug resistance, PTX, miR *let-7a*, Co-delivery nanosystem

## Abstract

**Background:**

Multidrug resistance (MDR) is the main challenge of successful chemotherapy for ovarian cancer patients, with 50% to 75% of ovarian cancer patients eventually relapsed due to it. One of the effective strategies for treating MDR and improving therapeutic efficiency of ovarian cancer is to use nanotechnology-based targeted drug delivery systems. In this study, a novel nano targeted co-delivery system modified by hyaluronic acid (HA) was developed by using gold nanorods coated with functionalized mesoporous silica nanoparticles (HA-PTX/*let-7a*-GNR@MSN) for combined delivery of hydrophobic chemotherapy drug Paclitaxel (PTX) and *lethal-7a* (*let-7a*), a microRNA (miR), to overcome MDR in ovarian cancer. Furthermore, we also analyzed the molecular mechanism of this nanotherapeutic system in the treatment of ovarian cancer.

**Results:**

HA-modified nanocomplexes can specifically bind to the CD44 receptor, which is highly expressed in SKOV3/SKOV3_TR_ cells, achieving effective cell uptake and 150% enhancement of tumor site permeability. The nanosystem realized the stable combination and protective transportation of PTX and miRs. Analysis of drug-resistant SKOV3_TR_ cells and an SKOV3_TR_ xenograft model in BALB/c-nude mice showed significant downregulation of P-glycoprotein in heterogeneous tumor sites, PTX release, and subsequent induction of apoptosis. More importantly, this nanosystem could synergistically inhibit the growth of ovarian tumors. Further studies suggest that mTOR-mediated signaling pathways play an important role in reversing drug resistance and inducing apoptosis.

**Conclusions:**

To sum up, these data provide a model for overcoming PTX resistance in ovarian cancer.

**Graphical Abstract:**

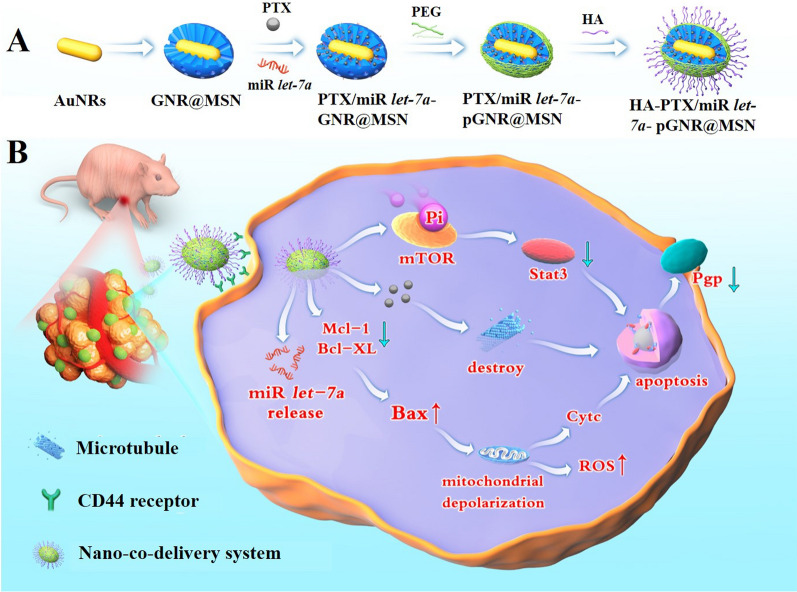

**Supplementary Information:**

The online version contains supplementary material available at 10.1186/s12951-021-01139-1.

## Background

Ovarian cancer is one of the most serious cancers that harm women’s health, with the highest morbidity and mortality among all gynecological malignancies [1, 2]. The main treatment strategy is surgery combined with chemotherapy based on paclitaxel (PTX), platinum, and other chemotherapy drugs. However, multidrug resistance (MDR) leads to poor effect or even failure of chemotherapy, and 50–75% of ovarian cancer patients eventually relapse because of MDR [3–6]. The high expression of MDR-related genes in ovarian tumor cells is the main cause of MDR in ovarian cancer [7, 8]. P-glycoprotein (P-gp), a 170 kDa transmembrane glycoprotein (also known as P-170) encoded by the MDR gene family and powered by adenosine triphosphate (ATP), continuously pumps chemotherapeutic drugs out of cancer cells, thereby reducing drug concentrations and cytotoxicity in cancer cells and ultimately leading to MDR. *MDR1* inhibitors can inhibit *MDR1* expression in cancer cells and reverse MDR [9, 10]. However, although *MDR1* inhibitors have been used for decades, their clinical effect is not sufficient enough to effectively reverse the MDR in ovarian cancer and improve prognosis. Therefore, it is very important to find a way to reverse MDR in ovarian cancer.

MicroRNAs (miRs) are small, endogenous single-stranded noncoding RNAs with a length of about 20–24 nucleotides. Some miRs, such as lethal 7 (let-7) and miR-199a-3p, may have an anti-ovarian cancer effect [11, 12]. However, these miRs are underexpressed in ovarian cancer cells, promoting cancer growth and anti-apoptosis, and weakening the killing effect of chemotherapy drugs on cancer cells. Therefore, exogenous supplementation of miRs, combined with chemotherapy drugs, shall enhance the anticancer effect and effectively reverse MDR. About 50% of miRs are located at vulnerable sites of cancer-related genomes. As an oncogene or tumor suppressor gene, miR plays an important role in cancer development and may become an important target for reversing MDR in ovarian cancer [13, 14]. The let-7 family is closely associated with the development of MDR in ovarian and other cancers [15]. Recent studies have shown that miR let-7 regulates the resistance of ovarian cancer cells to taxol by regulating IMP-1 mediated MDR1 stability [16]. The expression of *let-7a* in plasma and tissues of patients with ovarian cancer decreased significantly. Therefore, we hypothesize that exogenous supplementation of *let-7a*, combined with chemotherapeutic agents, can enhance the anticancer effect and reverse multidrug resistance in ovarian tumors. However, miR therapy has a potential risk of sequence specificity rather than gene specificity. Therefore, it is of great significance to improve the specificity and targeting of miR for ovarian cancer to improve the therapeutic effect and reduce adverse reactions. In addition, as miRNA is easy to degrade, it is difficult to effectively transfer it to the target. Therefore, it is very significant to develop a strategy to overcome the major hurdles facing the therapeutic miRNA delivery.

In recent years, targeted drug delivery systems based on nanotechnology have been widely used in the prevention, diagnosis, and treatment of various cancers [17–19]. Establishing a nanoplatform to co-deliver specific miRs and chemotherapy drugs to overcome MDR and effectively treat ovarian cancer might be a novel strategy. Among various nanobiomaterials, gold nanorods (GNRs) have shown great potential in cancer treatment because of their many advantages. Firstly, GNR preparation is simple to prepare and the particle size can be accurately controlled [20]. Secondly, the large specific surface area (SSA) of GNRs facilitates functional modification and further coupling of targeted ligands, such as receptor-targeted molecules, antibodies, and aptamers. Thirdly, GNRs have good stability and biocompatibility, so they can be used as an ideal carrier for drugs, genes, and nucleic acids [21–23]. However, the application of naked GNRs in drug delivery is limited because of their low drug loading, limited elasticity and cytotoxicity. In contrast, mesoporous silica nanoparticles (MSNs) are attracting increasing interest in gene/drug delivery because of their large pore size, high attraction, and favorable morphological properties [24]. Therefore, we used GNRs as miRNA/PTX delivery vehicle core, and further modified by MSNs with super-large pores which can provide the efficient attachment of miRNA/PTX, prevent the miRNA degradation, improve drug loading capability and cytotoxicity. The ligand HA was chosen, as HA was tumor-specific for receptor cluster of differentiation 44 (CD44), a membrane-anchored cell surface receptor usually detected in ovarian cancer cells, such as SKOV3 and SKOV3_TR_ cells. CD44 has been demonstrated to interact with HA at the N terminus of its extracellular domain, and therefore serves as a major cell surface receptor for ovarian cancer [25, 26].

**Scheme 1:**
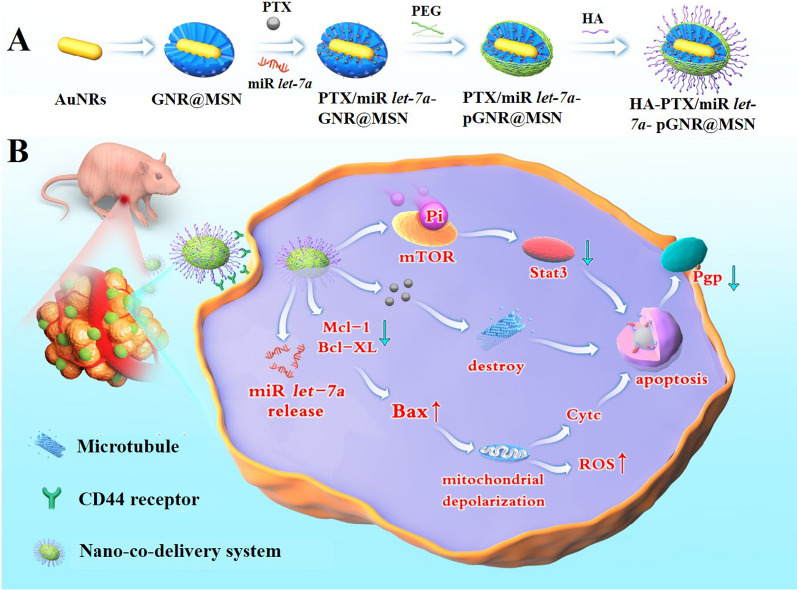
**A** Schematic illustration of the synthetic process for the HA-PTX/*let-7a*-GNR@MSN nano co-deliverysystem. **B** Serial targeted delivery and therapeutic mechanism of the HA-PTX/*let-7a*-GNR@MSN co-delivery nanosystem in the ovarian cancer SKOV3/SKOV3_TR_ cell line and tumor tissues. HA, hyaluronic acid; PTX, paclitaxel; miR, microRNA; *let-7a*, *lethal-7a*; GNR, gold nanorod; MSN, mesoporous silica nanoparticle; ROS, reactive oxygen species

In this study, a novel nano targeted co-delivery system modified by hyaluronic acid (HA) was developed employing GNRs coated with functionalized MSNs (GNR@MSN) (HA-PTX/*let-7a*-GNR@MSN) to co-deliver miR *let-7a* and PTX, a hydrophobic chemotherapy drug, to overcome MDR and and enhance the therapeutic efficiency of ovarian cancer treatment (Scheme 1).

## Materials and methods

### Materials

Gold chloride solution (HAuCl_4_) and tetraethyl orthosilicate (TEOS) were purchased from Aladdin Industrial Co., Ltd. (Shanghai, China); polyethylene glycol (NH_2_-PEG-COOH; molecular weight 3070) from Shanghai ZZBIO Co., Ltd. (Shanghai, China); fetal bovine serum (FBS) and Roswell Park Memorial Institute (RPMI) 1640 culture medium from Gibco (Invitrogen, Carlsbad, CA, USA); HA, acridine orange (AO), fluorescein diacetate (FDA), propidium iodide (PI), Hoechst 33258, 4ʹ,6-diamidino-2-phenylindole (DAPI), 3-(4,5-dimethylthiazlo-2-diphenyl-tetrazolium) bromide (MTT), Rhodamine B isothiocyanate (RBITC), PTX, and dimethyl sulfoxide (DMSO) were obtained from Sigma-Aldrich (St. Louis, MO, USA); an annexin V-fluorescein isothiocyanate (FITC) apoptosis detection kit from Keygen Biotech Co., Ltd. (Nanjing, China); Rhodamine 123 and 2ʹ-7ʹ dichlorofluorescin diacetate (DCFH-DA) from Solarbio Technology Co., Ltd. (Beijing, China). Monoclonal mouse anti-P-gp antibody, Horseradish peroxidase (HRP)-conjugated goat anti-rabbit IgG were obtained from Proteintech Group, Inc. (Chicago, IL, USA); other primary antibodies were purchased from Sigma-Aldrich (St. Louis, MO, USA). Other reagents and chemicals were of analytical grade unless otherwise stated and were purchased from local commercial suppliers. Deionized (DI) water (Milli-Q, Millipore, Bedford, MA, USA) was used to prepare aqueous solutions.

### Animals

About 4-week old male BALB/c-nu mice were obtained from Wuhan Laboratory Animal Co., (Wuhan, China) and were maintained under specific pathogen-free conditions. Animal experiments were conducted according to the guidelines specified by the Animal Care and Use Committee of Zhengzhou University (Zhengzhou, China).

### Gold nanorod synthesis

GNRs were synthesized using the seed growth method [21, 22]. First, gold seeds were synthesized using the chemical reduction method with HAuCl_4_ as the raw material and NaBH_4_ as the reducing agent. Next, gold nanoparticles (NPs) were formed in the solution using the gold seed-induced growth method. To prepare a seed solution, 125 µL of 1% HAuCl_4_ was added to cetyltrimethylammonium bromide (CTAB) (0.1 M, 7 mL) under stirring, followed by the addition of 300 µL of 0.01 M NaBH_4_. After the color of the solution changed from golden-yellow to brown, it was kept at 30 ℃ for 2 h. To prepare a growth solution, 7 mL of 0.1 M CTAB was mixed with 300 µL of 1% HAuCl_4_ solution, followed by the addition of 10 μL of 0.1 M AgNO_3_ under stirring. Then, 120 μL of 0.33 M hydroquinone was added, and the solution quickly became colorless. Finally, 100 μL of the seed solution was added, mixed, and allowed to be kept static at 30 ℃ for 12 h. The obtained GNRs were collected by centrifugation at 5000 rpm, room temperature (RT) for 3 min, dispersed in DI water, and stored at 4 ℃ for future use.

### GNR@MSN synthesis and amino modification

The GNRs synthesized were coated with MSNs using the Stober method. Briefly, the pH of the GNR solution (0.01 mM, 10 mL) was adjusted to 10–11 using 28 wt% NH_3_·H_2_O, followed by the addition of 17 μL of TEOS every 30 min four times in total. Subsequently, the reaction was performed at room temperature for 12 h. Then, the obtained GNR@MSN was rinsed by centrifugation with DI water and methanol at 5000 RPM at RT for 5 min, respectively.

For amino modification of GNR@MSN, the nanoparticles were dispersed in the mixture of 15 μL of 3-aminopropyltriethoxysilane (APTES) and 85 μL of methanol, and then refluxed at 75 ℃ for 6 h. Finally, the obtained GNR@MSN-NH_2_ were washed thrice with hot methanol by centrifugation at 5000 rpm for 10 min [27, 28].

### PTX and miR let-7a loading

PTX and miR *let-7a* were loaded onto GNR@MSN-NH_2_ through pore adsorption of mesoporous silica, where the mass ratio of PTX to GNR@MSN-NH_2_ was 1:1. Briefly, PTX was dissolved in 1 mL of DMSO and added to a solution of GNR@MSN-NH_2_. Then, the reaction proceeded overnight under continuous shaking [27]. Next, the obtained PTX-GNR@MSN were collected by centrifugation at 10,000 rpm for 10 min and dried under vacuum at 45 ℃ overnight. Subsequently, 0.25 mg PTX-GNR@MSN was dispersed in 40 μL of ethanol, followed by the addition of 10 μL of 4 M HCl and miR *let-7a* (20 μmol/L) for 2 h, the obtained PTX/miR *let-7a*-GNR@MSN were centrifuged at 10,000 rpm for 10 min [29]. The loading degrees of PTX and miR *let-7a* were determined by high-performance liquid chromatography (HPLC) and agarose gel electrophoresis, respectively, as shown below:

$${\text{Loading}}\hspace{0.33em}\text{content }(\%)=\frac{{W}_{\text{t}}}{{W}_{\text{s}}}\times 100\%,$$

$${\text{Encapsulation}}\hspace{0.33em}{\text{efficiency}}\hspace{0.33em}(\%)=\frac{{W}_{\text{t}}}{{W}_{0}}\times 100\%,$$

where *W*_t_ is the weight of PTX in NPs, *W*_s_ is the weight of NPs, and *W*_0_ is the initial weight of PTX in the system.

### Fabrication of PEG-modified GNR@MSN

PEG-modified GNR@MSN (pGNR@MSN) were fabricated using 1-ethyl-3-(3-dimethyllaminopro-pyl) carbodiimide hydrochloride/*N*-hydroxysuccinimide (EDC/NHS) coupling method. Briefly, 10 mg of NH_2_-PEG-COOH was dissolved in 8 mL of DMSO, followed by the addition of 38 mg of NHS and 68 mg of EDC dissolved in 2 mL of 2-(*N*-morpholino) ethanesulfonic acid (MES) buffer (pH 6.0). After the activation reaction proceeded at room temperature (RT) for 24 h, 10 mg GNR@MSN-NH_2_ was added and the reaction was kept at RT for another 24 h. Finally, the nanoparticles obtained by centrifuging the reaction mixture at 10,000 rpm for 10 min were washed with deionized water to remove excess PEG to obtain pGNR@MSN, which were dried under vacuum at 45 ℃ overnight and stored at 4 ℃ until use.

### Preparation of HA-modified nanocomposites

10 mg of HA was added to MES buffer (to pH 6.0) containing 5 mL EDC/NHS (2 mg/mL) for activation 1.5 h and then the pH of the solution was adjusted to 8.3 using Tris buffer. After the activation reaction proceeded for 12 h, 10 mg of pGNR@MSN were added and the reaction was continued at RT for 24 h. Finally, the nanoparticles obtained by centrifuging the reaction mixture at 10,000 RPM for 10 min were washed with methanol and deionized water to remove the impurities to obtain HA-pGNR@MSN, which were dried under vacuum 45 ℃ overnight and stored at 4 ℃ until use.

### Characterization

The morphology of the as-prepared pGNR@MSN was characterized employing high-angle annular dark-field scanning transmission electron microscopy (HAADF-STEM) and high-resolution transmission electron microscopy (HRTEM) using a JEM-2100 analytical electron microscope (JEOL Ltd., Tokyo, Japan). The crystal structure of the GNRs was measured using X-ray diffraction (XRD; X'Pert PRO MPD, Hol-land Panalytical) with a monochromatic X-ray beam and nickel-filtered Cu Ka radiation and the Fourier transform infrared (FT-IR) spectra of the pGNR@MSN were recorded using a FT-IR spectrometer (Nicolet IS50-Continuum; Thermo Fisher Scientific, Waltham, MA, USA). In addition, the N_2_ adsorption–desorption isothermal curve was recorded using the TriStar II 3020 system (Micromeritics, USA), and the zeta potential and particle size of the nanoparticles were determined using a Zetasizer Nano ZS90 analyzer (Malvern Panalytical, Malvern, Netherland).

### Cell culture

The ovarian cancer SKOV3 cell line was purchased from the Shanghai Cell Bank of the Chinese Academy of Sciences (Shanghai, China). SKOV3 cells were cultured in RPMI 1640 medium containing 10% FBS, 100 μg/mL of penicillin, and 100 μg/mL of streptomycin in a 5% CO_2_ humidified atmosphere at 37 ℃. The SKOV3_TR_ cell line (PTX-resistant SKOV3 cells) was constructed and maintained in RPMI 1640 medium containing 10 μL of PTX (2 mg/mL).

### Hemolysis assay

The blood compatibility of HA-pGNR@MSN was evaluated by hemolysis assay. Briefly, fresh human blood was centrifuged at 3000 rpm for 15 min and the supernatant was removed, followed by washing with 0.9% saline solution to obtain red blood cells (RBCs). Next, a proper amount of 0.9% saline was added to the RBCs to prepare a suspension contaiing 2% RBC and 1 mL of such suspension was then added to the solution of HA-pGNR@MSN at different concentrations (50–400 μg/mL) with saline and DI water as negative and positive controls, respectively. The samples were stabilized at RT for 2 h and then centrifuged at 3000 rpm. Finally, the supernatant was collected and measured absorbance at the wavelength of 540 nm to obtain the image of the sample and calculate the hemolysis rate.

### in vitro cytotoxicity assay

The cytotoxicity of HA-pGNR@MSN was detected using 3-(4,5-dimethylthiazol-2-yl)-2,5- diphenyltetrazolium bromide (MTT) assay. Briefly, SKOV3 cells were seeded on a 96-well plate at a density of 3000 cells/well and incubated for 12 h. Next, a series of HA-pGNR@MSN solutions at different concentrations (0, 25, 50, 100, 200, and 400 μg/mL) were added to the 96-well plate. After incubation at 37 ℃ for 24 h, the medium was removed and 0.5 mg/mL of MTT solution (200 μL/well), was added, followed by incubation for another 4 h. Subsequently, DMSO was added (150 μL/well) and the mixture was vortexed. Finally, the absorbance of the sample was measured at the wavelength of 570 nm using a microplate spectrophotometer (BioTek Instruments Inc., Winooski, VT, USA) [30].

### RBITC-labeled nanocomposites for cell uptake analysis

Cell uptake analysis was performed using fluorescent RBITC-labeled nanocomposites. Briefly, HA-pGNR@MSN and pGNR@MSN were labeled using fluorescent RBITC via the amino groups on the surface of the nanoparticles [31]. Next, SKOV3 cells were seeded on a 24-well plate and incubated for 48 h, followed by the addition of fluorescent RBITC-labeled HA-pGNR@MSN and pGNR@MSN, followed by incubatiton in the dark for another 4 h. The cells were then rinsed thrice with PBS and photographed under an inverted EclipseTE2000-U fluorescence microscope (Nikon, Japan). RNA transfection efficiency was detected employing carboxyfluorescein (FAM)-labeled miR *let-7a*, whose uptake by SKOV3_TR_ cells was performed as mentioned earlier.

### Analysis of antiproliferation effects

The anti-proliferation ability of various therapeutic nanocomposites was examined using MTT assay and cell viability was assessed using an FDA and PI double staining method [32]. Briefly, SKOV3 and SKOV3_TR_ cells treated with various therapeutic nanocomposites were washed with PBS and incubated with 1 μg/mL of FDA and 20 μg/mL of PI for 5 min. The cells were then washed with PBS and photographed under an inverted Eclipse TE2000-U fluorescence microscope.

### Apoptosis assay

Hoechst 33258 was used to detect the apoptosis of SKOV3 and SKOV3_TR_ cells treated with various therapeutic nanocomposites [33]. Briefly, SKOV3 and SKOV3_TR_ cells were cultured on a 24-well plate for 12 h, followed by the addition of HA-miR *let-7a*-GNR@MSN, HA-PTX-GNR@MSN, PTX/miR let-7a-GNR@MSN, and HA-PTX/miR *let-7a*-GNR@MSN, respectively. After treatment for 24 h, the drug and the original culture solution were removed and the treated cells were rinsed with PBS once. Next, 500 μL of 0.5 mg/mL Hoechst 33258 staining solution was added to each well, followed by incubation in the dark for 20 min. The cells were then washed with PBS twice to remove the unreacted staining solution and observed under an inverted Eclipse TE2000-U fluorescence microscope.

Annexin V-FITC/PI double-staining assay was used to detect the apoptosis of treated SKOV3 and SKOV3_TR_ cells. Briefly, the treated SKOV3 and SKOV3_TR_ cells were digested using 0.25% trypsin and then centrifuged at 1000 rpm for 5 min for collection. Next, the cells collected were resuspended in 500 μL of PBS, followed by the addition of 5 μL of annexin V-FITC and PI, respectively. Finally, the mixture was incubated in the dark at RT for 10 min and then the cells were analyzed using FACS Calibur flow cytometry (BD Biosciences, San Jose, CA, USA) and Cell-Quest software (BD Biosciences).

### Mitochondrial membrane potential detection

Mitochondrial membrane potential (MMP) was detected by Rhodamine 123 staining. Briefly, SKOV3 and SKOV3_TR_ cells were treated with various therapeutic nanocomposites for 24 h, washed with PBS twice, stained with 50 μg/mL of Rhodamine 123, and incubated in the dark for 30 min. Next, the cells were again washed with PBS twice and photographed under an inverted EclipseTE2000-U fluorescence microscope.

### Analysis of reactive oxygen species

SKOV3 and SKOV3_TR_ cells treated with various therapeutic nanocomposites were washed twice with PBS, treated with 50 μM DCFH-DA in FBS-free medium, and incubated in the dark for 15 min. Next, the cells were again washed with PBS twice, and reactive oxygen species (ROS) were analyzed using FACS Calibur flow cytometry (BD Biosciences, San Jose, CA, USA) and Cell-Quest software (BD Biosciences).

### Acridine orange staining

SKOV3 and SKOV3_TR_ cells treated with various therapeutic nanocomposites for 24 h were washed with PBS twice and then resuspended in 300 μL of PBS. Next, 300 μL of 0.01% AO staining solution was added, followed by incubation the dark at RT for 15 min. Next, the cells were washed with PBS twice and photographed under an inverted fluorescence Eclipse TE2000-U microscope.

### Western blotting

SKOV3 and SKOV3_TR_ cells treated with various therapeutic nanocomposites were lysed in lysis buffer (50 mM Tris-HCl [pH 7.4], 150 mM NaCl, 1% NP-40, 0.5% sodium dexoycholate, 0.1% sodium dodecyl sulfate [SDS], 2 mM phenylmethylsulfonyl fluoride [PMSF]) and then boiled for 5 min in a metal bath at 100 °C. Next, the total protein extracts (10 μg) were separated employing 12% sodium dodecyl sulfate-polyacrylamide gel electrophoresis (SDS-PAGE) and then transferred onto a polyvinylidene difluoride (PVDF) membrane (Merck Millipore, Burlington, MA, USA) blocked with 8% (w/v) nonfat milk in 0.05% phosphate-buffered saline-Tween 20 (PBST) buffer for 1 h. The PVDF membrane was washed with PBST buffer and then incubated overnight with primary antibodies (1:5000 or 1:10,000 in PBST) at 4 ℃ and appropriate horseradish peroxidase (HRP)-conjugated secondary antibodies (1:10,000 or 1:20,000) for 1 h at RT. Finally, immunoreactive bands were developed using Pierce ECL Western Blotting Substrate (Thermo Fisher Scientific), and the relative protein quantity was normalized to the level of glyceraldehyde 3-phosphate dehydrogenase (GADPH).

### Establishment of SKOV3_TR_ tumor xenograft model

Four-week-old male BALB/c-nu mice were obtained from Wuhan Laboratory Animal Co., (Wuhan, China) and maintained under the specific pathogen-free (SPF) condition. SKOV3_TR_ cells at the logarithmic growth stage were used to prepare 100 μL cell suspension (density: 8 × 10^6^ cells). The suspension was inoculated subcutaneously on the dorsal side of mice. The health status and behavior of the mice were observed every day and the tumor volume and body weight were recorded every 2 days. The tumor volume was calculated as follows:

$$V = {1}/{2} \times a \times b^{{2}} ,$$

where *a* and *b* are the long and short diameters of the tumor, respectively. The tumor suppression rate was calculated as follows:

$$R\left( \% \right) = ({1}{-}V_{{\text{t}}} /V_{0} ) \times {1}00\% ,$$

where *V*_t_ and *V*_0_ are the tumor volume of the treatment and the control group, respectively. When the average tumor volume reached 30 mm^3^, various therapeutic nanocomposites prepared for drug test were injected.

### Drug intervention and histopathological analysis

In the successfully established SKOV3_TR_ tumor xenograft model, the BALB/c-nu mice were randomly divided into five groups and treated with various therapeutic nanocomposites: HA-miR *let-7a*-pGNR@MSN, HA-PTX-pGNR@MSN, PTX/miR *let-7a*-pGNR@MSN, and HA-PTX/miR let-7a-pGNR@MSN, with 0.9% saline as control. Intratumoral injection was performed using the whole constructive nanocomposites at a rate of 15 mg/kg (200 μL) every 2 days [34, 35]. After treatment, the mice were euthanized, and the tumors and main organs (liver, heart, lungs, kidneys, and spleen) were collected for histopathological analysis by hematoxylin and eosin (H&E) staining, terminal deoxynucleotidyl transferase dUTP nick end labeling (TUNEL) staining, and antigen Ki-67 staining. Finally, the corresponding tumor sites were analyzed by western blotting to detect the expression of P-gp.

### Statistical analysis

All the data were presented as mean ± standard deviation (SD). Student’s *t*-test was used for statistical comparison. *p < 0.05 was considered significant, **p < 0.01 was considered moderately significant) and ***p < 0.001 was considered highly significant.

## Results and discussion

### Synthesis and characterization of HA-pGNR@MSN

Herein, we report a novel HA-modified targeted nano drug delivery system that uses functionalized mesoporous silica nanoparticle-coated gold nanorods (HA-GNR@MSN) to co-deliver PTX and miR *let-7a* to overcome MDR in ovarian cancer.

The synthesis procedure of the targeted nanosystem is shown in Scheme 1, which includes: (i) synthesis of GNRs utilizing improved seed growth method; (ii) encapsulation of GNR with mesoporous silicon shells with large aperture to to load PTX and miR *let-7a* by aperture adsorption and electrostatic shielding method; (iii) amine modification with APTES; (iv) loading PTX and miR *let-7a* into silicon pores; (v) modification with NH_2_-PEG-COOH (to ensure the blood circulation time and subsequent modification ability of the nanoparticles); and (vi) conjugation of this nanocomposite with HA via EDC/NHS to forme a specifically targeted Nano delivery system (HA-PTX/*let-7a-*GNR@MSN) to effectively enhance ovarian cancer treatment through multidrug resistance reversal.

**Fig. 1:**
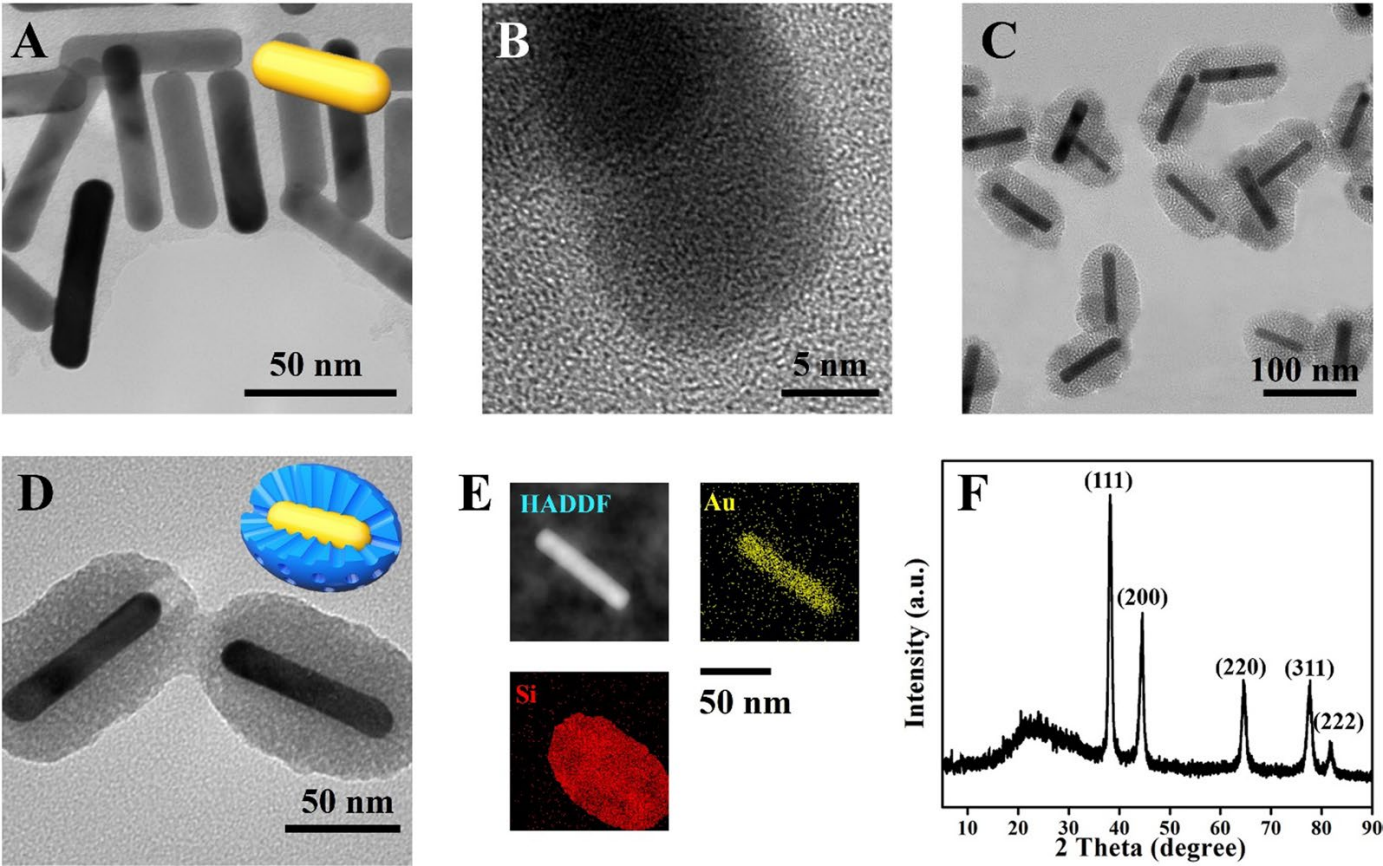
Characterization of GNR and GNR@MSN. **A** TEM and **B** HRTEM images of GNRs, **C** TEM, **D** partial enlarged TEM, **E** HAADF and EDS mapping images of GNR@MSN. **F** XRD patterns of GNRs. GNRs, gold nanorods; GNR@MSN, mesoporous silica nanoparticle-coated gold nanorods; TEM, transmission electron microscopy; HRTEM, high-resolution transmission electron microscopy; HAADF, high-angle annular dark-field; EDS, energy-dispersive X-ray spectroscopy; XRD, X-ray diffraction

The as-prepared nanoparticle complexes were characterized subsequently. Transmission electron microscopy (TEM) showed that the average length and width of the prepared GNRs were 75 ± 5 and 5 ± 5 nm, respectively (~ 7:1 aspect ratio) (Fig. 1A). Further measurement of the GNRs by HRTEM showed clearly visible bright and dark stripes of the lattice spacing (Fig. 1B). In addition, TEM of the obtained GNR@MSN core–shell NPs indicated that the GNRs were successfully coated by mesoporous silicon, and the average diameter of GNR@MSN was 135 ± 5 nm (Fig. 1C, D). In HAADF mode, analysis of GNR@MSN using a dark-field detector and energy-dispersive X-ray spectroscopy (EDS) element mapping showed that the GNRs were mainly distributed in the brighter region. In addition, the two elements (Au, Si) in GNR@MSN were also distributed in the brighter region, as expected (Fig. 1E). The diffraction peaks of the GNRs appeared at 2*θ* of 38.2°, 44.4°, 64.6°, 77.6°, and 81.7°, which were correlated with the lattice planes (111), (200), (220), (311), and (222) of Au, respectively (Fig. 1F).

**Fig. 2:**
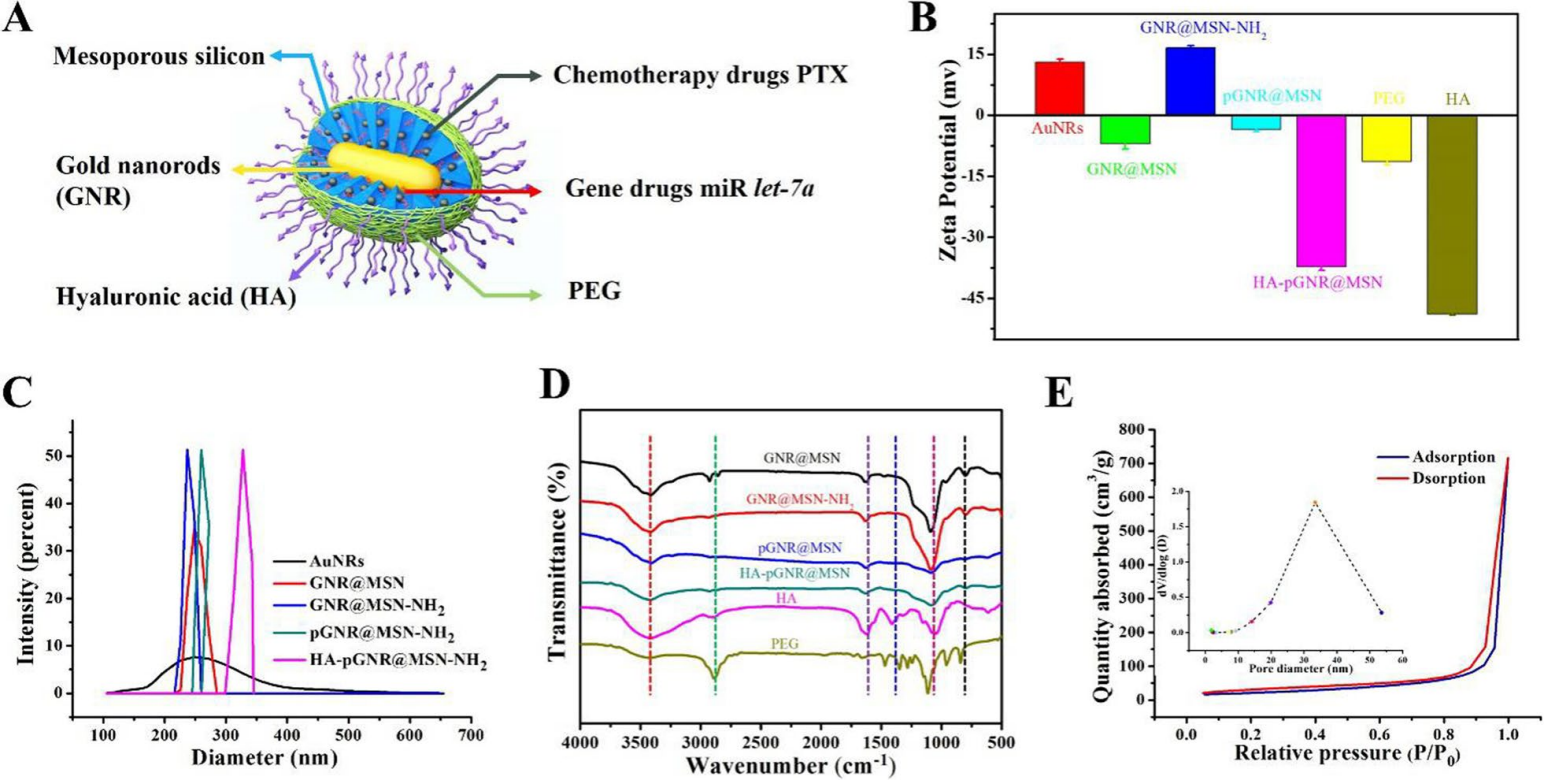
Characterization of HA-pGNR@MSN. **A** Structure of HA-pGNR@MSN. **B** Zeta potential assay of GNRs and various therapeutic nanocomposites. **C** Diameter distribution of GNRs and various therapeutic nanocomposites. **D** FT-IR spectra of GNRs, GNR@MSN, GNR@MSN-NH_2_, pGNR@MSN, and HA-pGNR@MSN. **E** N_2_ absorption–desorption isotherm of GNR@MSN and corresponding pore size distribution curve (inset). HA, hyaluronic acid; pGNR, polyethylene glycol-modified gold nanorod; MSN, mesoporous silica nanoparticle; FT-IR, Fourier transform infrared

The detailed structure of HA-modified nanocomposites was shown in Fig. 2A. The zeta potential changes of various therapeutic nanocomposites (+ 13.00, − 6.94, + 21.70, − 3.55, and − 37.33 mv) confirmed the successful modification of each component (Fig. 2B). The hydrodynamic diameter distributions of the prepared NPs were 255.7, 398.7, 488.9, 802.2, and 929.7 nm respectively, corresponding to GNRs, GNR@MSN, GNR@MSN-NH_2_, pGNR@MSN, and HA-pGNR@MSN (Fig. 2C). The obtained hydrated particle size data may be caused by polyhydroxy polymer compounds such as polyethylene glycol (PEG) and HA.

Further, FT-IR spectroscopy was used to detect the structure of the nanocomposites. The FT-IR spectrum of GNR@MSN-NH_2_ contained signals associated with N–H (1560 cm^−1^), indicating that the amination of GNR@MSN was successful. Owing to the modification of the MMSN surface with HA, the characteristic bands of amide bond were identified at the amide N–H stretching (3680 cm^−1^) and amide C=O stretching (1690 cm^−1^). In addition, the vibrational absorption peaks of Si–O–Si in GNR@MSN appear at 1081 cm^−1^ and 798 cm^−1^. The pore size and SSA of GNR@MSN were determined by the N_2_ absorption–desorption technique using Barrett–Joyner–Halenda (BJH) and Brunauer–Emmett–Teller (BET) methods. The SSA was high (77.00 m^2^/g) and the average pore size was 34.22 nm (Fig. 2E), indicating that the pores of mesoporous silica could effectively adsorb PTX and miR *let-7a*.

### Evaluation of biosafety, stability and fluorescent properties of nanocomposites

**Fig. 3:**
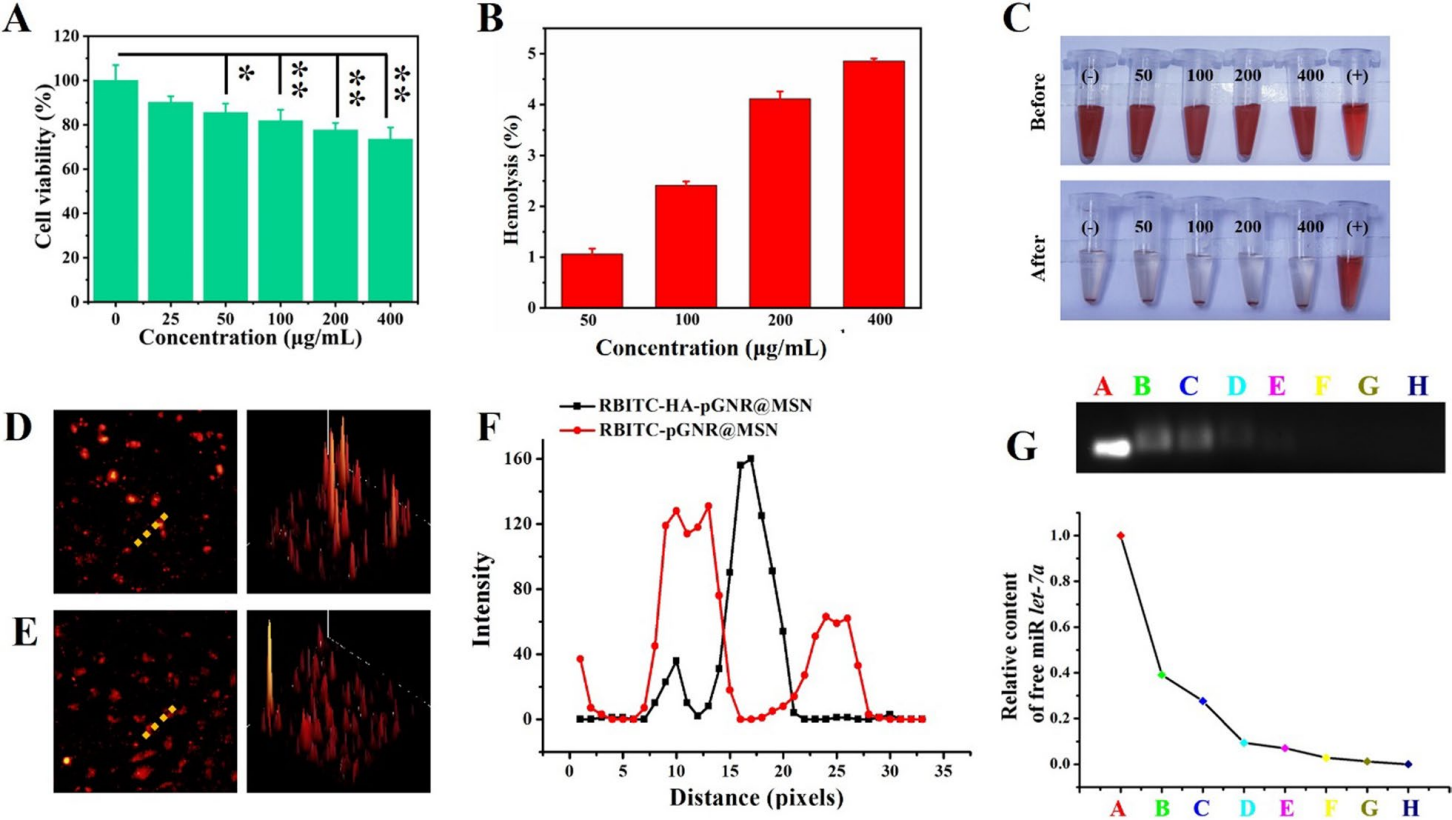
Assessment of biosafety, fluorescent properties, and drug loading of the HA-pGNR@MSN nano co-deliverysystem. **A** Cell vitality after 24 h treatment with HA-pGNR@MSN at various concentrations. **B** Hemolysis of HA-pGNR@MSN. **C** Digital photographs after incubation with RBCs at various concentrations for 2 h. **D** Fluorescent RBITC-labeled HA-pGNR @MSN and **E** pGNR@MSN. **F** Corresponding fluorescence profiling graph analyzed with Image-Pro Plus software. **G** Visualization of gel retardation assay of HA-pGNR@MSN binding with *let-7a* at different volume HA-pGNR@MSN:*let-7a* ratios: 0, 40:1, 80:1, 120:1, 160:1, 200:1, 240:1, and 280:1 from A to H, respectively. HA, hyaluronic acid; pGNR, NH_2_-PEG-COOH-modified gold nanorod; MSN, mesoporous silica nanoparticle; PTX, paclitaxel; *let-7a*, *lethal-7a*; RBC, red blood cell; RBITC, Rhodamine B isothiocyanate

Low cytotoxicity is crucial to construct HA-pGNR@MSN nano co-delivery system. First, the cytotoxicity of HA-pGNR@MSN at different concentrations was assessed. SKOV3 cells showed no significant cytotoxicity after treatment with 0, 25, 50, 100, 200, and 400 μg/mL of HA-pGNR@MSN for 24 h (Fig. 3A).

Hemocompatibility is another vital issue in blood-contacting applications of NPs [28]. Therefore, the hemolysis of HA-pGNR@MSN before use for gene/drug delivery was examined. Although the concentration of HA-pGNR@MSN reached 400 μg/mL, the hemolysis rate was only 4.85% (< 5%), (Fig. 3B, C), indicating that HA-pGNR@MSN could be used for subsequent in vivo and in vitro experiments. The stability of the prepared HA-pGNR@MSN in different media including 1640 medium, PBS (pH 7.4), and DI water was presented in Additional file 1: Fig. S2A, indicating the composites had excellent stability in different media. Besides, RBITC-labeled NPs showed strong red fluorescence, indicating that they could be effectively used subsequent cell uptake analysis (Fig. 3D–F).

### Drug loading and release of the HA-PTX/*let-7a*-GNR@MSN nano co-delivery system

Hydrophobic drugs are difficult to bind to NPs [26]. In addition, as RNA is easy to degrade, it is difficult to effectively transfer it to the target [27]. Therefore, GNR was modified by MSN with super-large pores to prepare PGNR for drug adsorption. The PEG of pGNR@MSN can prolong the blood circulation time of a drug. In addition, the targeted ligand HA can specifically distinguish the CD44 receptor, which is highly expressed in ovarian cancers SKOV3/SKOV3_TR_. Therefore, the administration efficiency can be improved and the therapeutic effect can be further improved.

The loading degree of PTX was determined by HPLC. According to the peak area and standard curve equation of PTX in Additional file 1: Fig. S1A, B, the standard equation was $y=-7.8\times 1{0}^{5}+5.1\times {10}^{5}x$ (*r*^2^ = 0.9909) and the drug loading was 3.82%.

In addition, the controlled release of PTX from HA-PTX/ miR *let-7a*-GNR@MSN was evaluated in the PBS solution with representative pH including (pH 5.0 and 7.4) (Additional file 1: Fig. S2B), and the results showed that the release of free PTX much faster than loading onto HA-PTX/miR *let-7a*-GNR@MSN at pH 5.0 and 7.4, approximately 90.7% (pH 5.0) and 78.6% (pH 7.4) was released within 12 h, 64.3 and 43.7% of PTX was released from HA-PTX/miR *let-7a*-GNR@MSN during the 72 h at pH 5.0 and pH 7.4. The sustained release of PTX from HA-PTX/miR *let-7a*-GNR@MSN could be beneficial for enhancing long-term antitumor efficiency and improving drug accumulation at a targeted site. These data also show the release of PTX is more rapidly in the mildly acidic microenvironment in the tumor area than in normal tissue or blood. PTX, as a classical clinical anti-tumor drug, can disbalance the synthesis and depolymerization of tubulin, induce and promote tubulin polymerization and microtubule assembly, and trigger cell apoptosis, thereby effectively preventing the proliferation of ovarian cancer cells.

In order to obtain the optimal binding ratio of miR *let-7a* and nanocarrier, agarose gel electrophoresis assay was performed. The final concentration of miR *let-7a* was 20 μM, and a series of proportional gradiations of GNR@MSN-NH_2_ and miR *let-7a* were set as 40:1, 80:1, 120:1, 160:1, 200:1, 240:1, and 280:1 (w/w). Next, gel block was used to determine the optimal ratio. At the ratio of 200:1, no free *let-7a* was found in the supernatant (Fig. 3G), indicating that miR *let-7a* was completely bound to GNR@MSN-NH_2_ at this ratio. In the following experiment, the ratio of 200 μg:1 μg (GNR@MSN-NH_2_:miR *let-7a*) was used for subsequent drug intervention experiments.

For cellular uptake assay, RBITC-labeled NPs can be effectively endocytosed via targeted HA, and they effectively recognize and bind to the CD44 receptor, which is highly expressed in SKOV3/SKOV3_TR_ cells, thus improving cell uptake efficiency. The cellular uptake efficiency of HA-pGNR@MSN increased ~ 300% times than pGNR@MSN (Additional file 1: Fig. S3A, B). Fluorescent double-labeled HA-^FAM^miR let-7a-^RBITC^pGNR@MSN was used to further confirm the transfection effeciency of the NPs after co-incubation with SKOV3_TR_ cells for 6, 12, and 24 h. The result demonstrated that the targeted HA-pGNR@MSN more effectively delivered miR let-7a to SKOV3/SKOV3_TR_ cells, and the maximum transfection rate was achieved 12 h post treatment (Additional file 1: Fig. S3C).

### Antiproliferative and therapeutic effects of nanocomposites in vitro

**Fig. 4:**
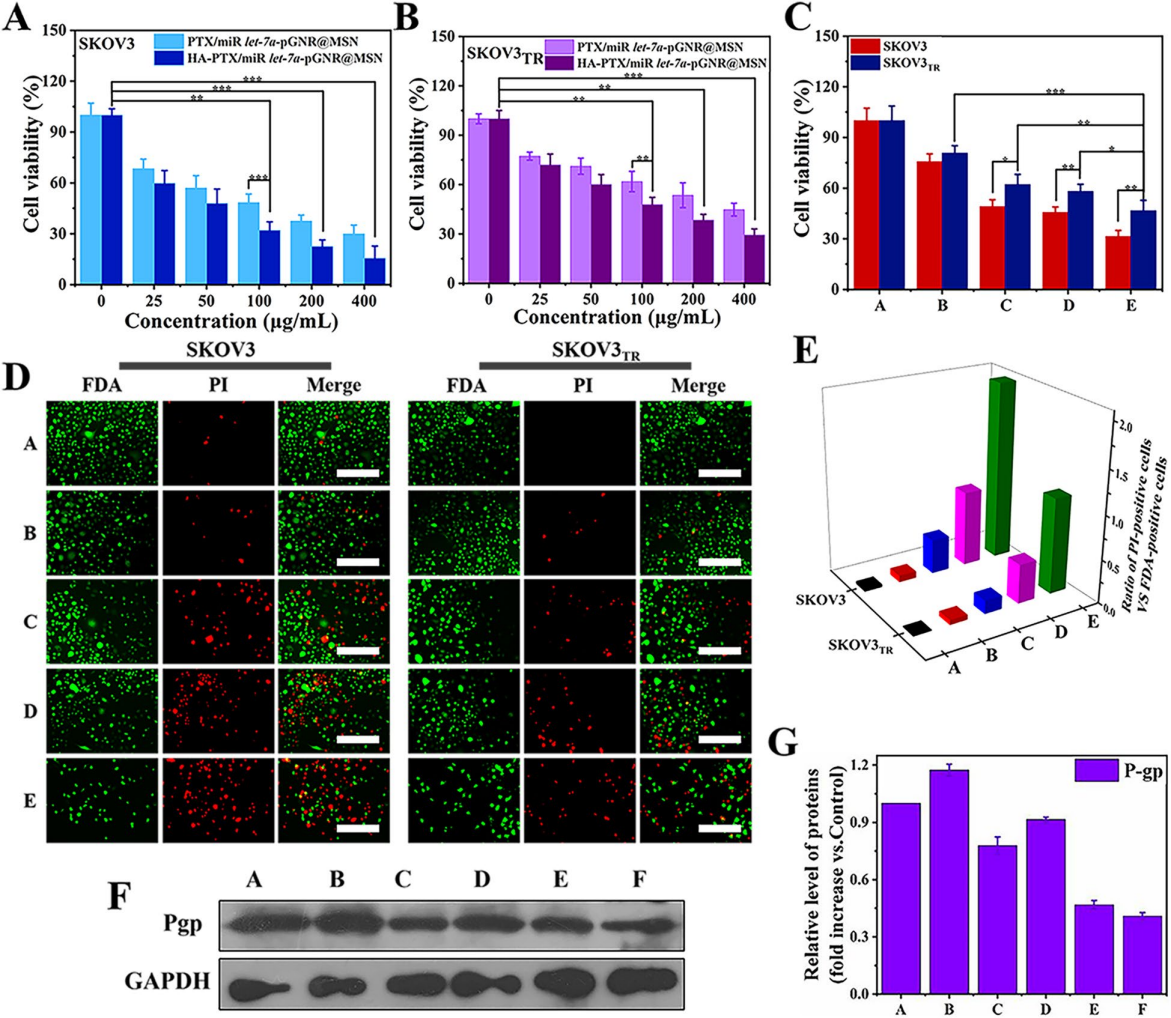
Evaluation of the antiproliferative ability of the therapeutic nanocomposites. **A** Cell viability of SKOV3 and **B** SKOV3_TR_ cells treated with targeted HA-PTX/*let-7a*-pGNR@MSN and nontargeted PTX/*let-7a*-pGNR@MSN. **C** Therapeutic effects of various therapeutic nanocomposites on SKOV3_TR_ cell proliferation: A, control; B, HA-miR *let-7a*-pGNR@MSN; C, HA-PTX-pGNR@MSN; D, PTX/*let-7a*-pGNR@MSN; E, HA-PTX/miR *let-7a*-pGNR@MSN. **D** Cellular viability assay using a PI and FDA double-staining protocol and **E** corresponding quantitative analysis of 24 h treatment with 100 μg/mL of various therapeutic nanocomposites: A, Control; B, HA-*let-7a*-pGNR@MSN; C, HA-PTX-pGNR@MSN; D, PTX/miR *let-7a*pGNR@MSN; E, HA-PTX/miR *let-7a*-pGNR@MSN. Scale bar = 200 μm. **F** Western blotting was used to measure P-gp expression after 24 h treatment with 100 μg/mL of various therapeutic nanocomposites and **G** corresponding quantitative analysis: A, control (SKOV3 cells); B, control (SKOV3_TR_ cells); C, HA-miR *let-7a*-pGNR@MSN; D, HA-PTX-pGNR@MSN; E, PTX/miR *let-7a*-pGNR@MSN; F, HA-PTX/miR *let-7a*-pGNR@MSN. HA, hyaluronic acid; PTX, paclitaxel; *let-7a*, *lethal-7a*; pGNR, polyethylene glycol-modified gold nanorod; MSN, mesoporous silica nanoparticle; FDA, fluorescein diacetate; PI, propidium iodide; P-gp, P-glycoprotein

MTT assay was used to evaluate the anti-proliferative ability of the therapeutic nanocomposites. Compared to PTX/miR *let-7a*-GNR@MSN, HA-PTX/miR *let-7a*-GNR@MSN showed a better therapeutic effect on SKOV3 cells (Fig. 4A). However, treated SKOV3_TR_ cells showed less effect than SKOV3 cells (Fig. 4B). This may be due to the high P-gp expression in SKOV3_TR_ cells, which can induce the active excretion of some drugs. However, the proliferation ability of SKOV3_TR_ cells treated with HA-PTX/miR *let-7a*-pGNR@MSN was significantly lower than that of SKOV3_TR_ cells treated with PTX/miR *let*-*7a*-pGNR@MSN (Fig. 4C). In addition, the HA-PTX/miR *let-7a*-GNR@MSN nano co-delivery system enhanced therapeutic efficacy, which can be attributed to the target ability of hyaluronic acid to the highly expressed CD44 protein receptor on the surface of SKOV3/SKOV3_TR_ cells. (Fig. 4C). As shown in Fig. 4D, E, FDA/PI double-staining analysis showed that HA-PTX/let-7a-pGNR@MSN had a better therapeutic effect than PTX/*let-7a*-pGNR@MSN.

Western blotting was also used to detect the expression of P-gp in treated SKOV3_TR_ cells. It can be seen from Fig. 4F that the expression of P-gp in the HA-miR *let-7a*-pGNR@MSN group was lower than that in the HA-PTX-pGNR@MSN group, indicating that *let-7a* could effectively reduce the expression of P-gp, thus further reducing the drug resistance of SKOV3_TR_ cells to PTX. In addition, the expression of P-gp in the HA-PTX/miR *let-7a*-pGNR@MSN group was the lowest, indicating that miR *let-7a* further enhanced the chemotherapy effect of PTX and promoted the apoptosis of SKOV3_TR_ cells. As shown in Additional file 1: Fig. S4A, B, the expression of P-gp decreased significantly with the increase of HA-PTX/miR *let-7a*-pGNR@MSN concentration, mainly because the therapeutic nanocomposites reversed MDR in SKOV3_TR_ cells.

### Analysis of the apoptosis mechanism

**Fig. 5:**
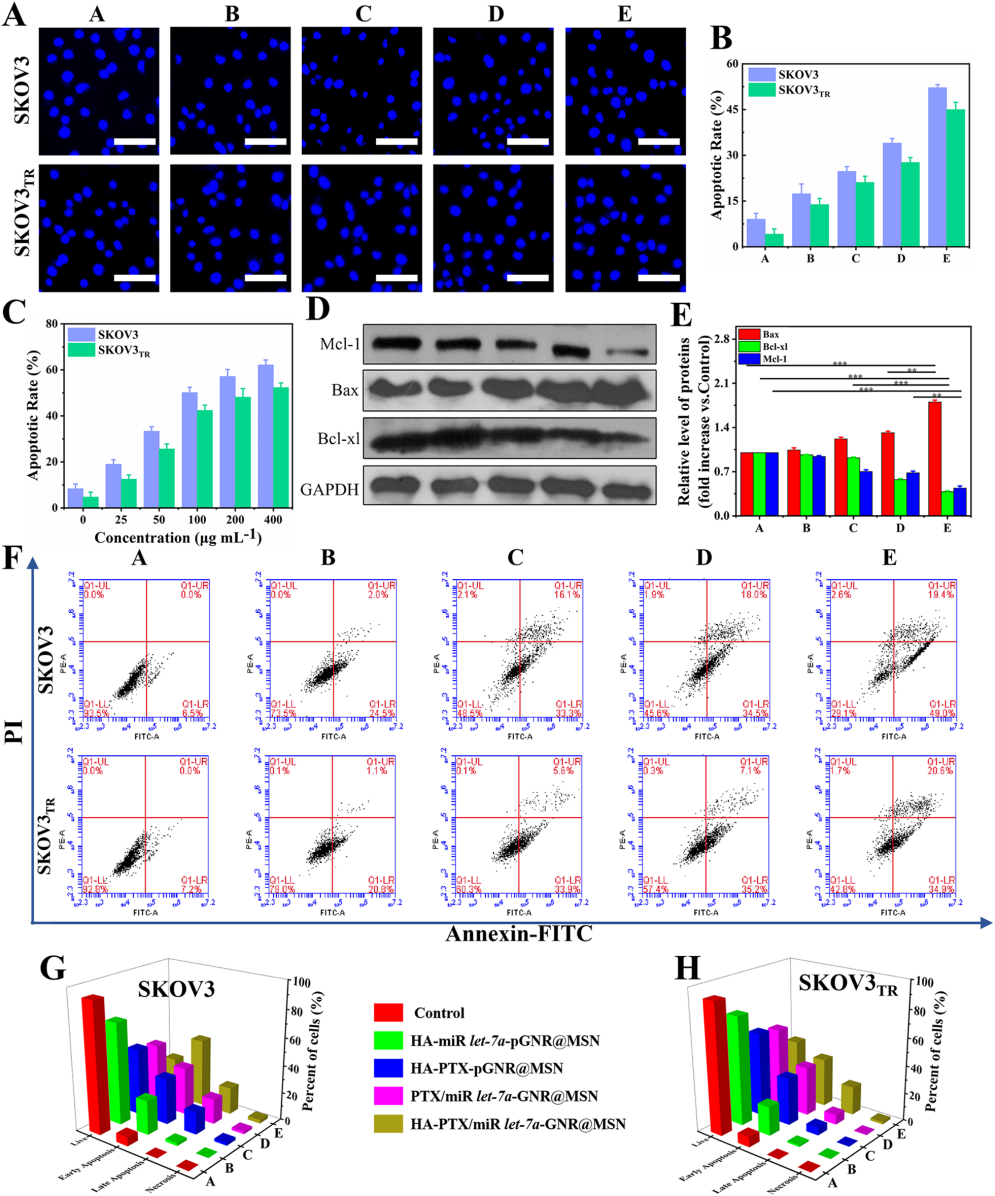
Apoptosis analysis of treated SKOV3 and SKOV3_TR_ cells. **A** Fluorescence images using Hoechst H33258 staining and **B** corresponding quantitative analysis of apoptotic rate of SKOV3 and SKOV3_TR_ cells treated with various therapeutic nanocomposites: A, control; B, HA-miR let-7a-pGNR@MSN; C, HA-PTX-pGNR @ MSN; D, PTX/miR let-7a pGNR@MSN; E, HA-PTX/miR let-7a-pGNR@MSN. Scale bar = 100 μm. **C** Analysis of apoptotic rate of SKOV3 and SKOV3_TR_ cells treated with various concentrations of HA-PTX/miR let-7a-pGNR@MSN (0, 25, 50, 100, 200, and 400 μg/mL). **D** Western blotting of apoptosis-associated proteins in SKOV3_TR_ cells treated with various therapeutic nanocomposites and **E** statistical analysis of relative protein levels: A, control; B, HA-miR let-7a-pGNR@MSN; C, HA-PTX-pGNR@MSN; D, PTX/miR let-7a pGNR@MSN; E, HA-PTX/miR let-7a-pGNR@MSN. **F** Flow cytometric analysis of apoptosis using annexin V-FITC/PI staining of SKOV3 and SKOV3_TR_ cells after treatment with various therapeutic nanocomposites. A, control; B, HA-miR let-7a-pGNR@MSN; C, HA-PTX-pGNR@MSN; D, PTX/miR let-7a pGNR@MSN; E, HA-PTX/miR let-7a-pGNR@MSN. **G**, **H** Corresponding quantification analyses of the percentage of live, early apoptotic, late apoptotic, and necrotic cells after treatment of SKOV3 and SKOV3_TR_ with various therapeutic nanocomposites: A, control; B, HA-miR let-7a-pGNR@MSN; C, HA-PTX-pGNR@MSN; D, PTX/miR let-7a-pGNR @MSN; E, HA-PTX/miR *let-7a*-pGNR@MSN. HA, hyaluronic acid; *let-7a*, *lethal-7a*; pGNR, polyethylene glycol-modified gold nanorod; MSN, mesoporous silica nanoparticle; PTX, paclitaxel; FITC, fluorescein isothiocyanate; PI, propidium iodide

In order to observe the nuclear division of apoptotic cells, treated SKOV3 and SKOV3_TR_ cells were stained with Hoechst H33258, a fluorescent dye that binds to AT-rich DNA regions [30]. The typical changes in SKOV3 and SKOV3_TR_ apoptotic cells after treatment were chromatin condensation, perinuclear aggregation, and nuclear fragmentation (Fig. 5A, B). In addition, nuclear fragmentation and chromatin condensation were significantly increased with the increase of HA-PTX/miR *let-7a*-pGNR@MSN concentration (Additional file 1: Fig. S5 and Fig. 5C).

Myeloid leukemia factor-1 (Mcl-1), an anti-apoptotic member of the B-cell lymphoma 2 (Bcl-2) family, is highly expressed in cancer cells [36]. The expression of Mcl-1 not only promotes the generation and development of tumors, but also leads to the drug resistance of cancer cells to chemotherapeutic drugs. As shown in Fig. 5D, E, the expression of in the HA-PTX/miR *let-7a*-pGNR@MSN group was significantly reduced, indicating that the sensitivity of SKOV3_TR_ cells were significantly more sensitive to PTX and further induced apoptosis. The expression of Mcl-1 also decreased with the increase of HA-PTX/miR let-7a-pGNR@MSN concentration (Additional file 1: Fig. S6A, B). In addition, the expression of pro-apoptotic Bcl-2-associated X protein (Bax) was increased in SKOV3_TR_ cells in the HA-PTX/miR *let-7a*-pGNR@MSN group compared to other groups, while the expression of anti-apoptotic B-cell lymphoma extra large (Bcl-XL) was decreased, indicating that MDR in SKOV3_TR_ cells can be reversed.

Annexin V-FITC and PI staining were used to further detect the mechanism of MDR reversal. According to fluorescence intensity, the treated cells were divided into four quadrants: living cells, early apoptotic cells, late apoptotic cells and necrotic cells (Fig. 5F). Figure 5G, H show show a quantitative analysis of the number of cells in the four quadrants of each group. The survival rates of SKOV3 and SKOV3_TR_ cells were 29.1% and 42.8% after treatment with HA-PTX/miR *let-7a*-pGNR@MSN for 24 h, respectively, indicating that HA-PTX/miR *let-7a*-pGNR@MSN significantly affected cell apoptosis. In addition, the number of early apoptotic cells was significantly higher than that of late apoptotic cells, which may be due to the reversal of phosphatidylserine (PS) from the interior to the surface of the cell membrane and exposure to the extracellular environment.

### Effects of therapeutic nanocomposites on related organelles and mTOR-mediated signaling pathways

**Fig. 6:**
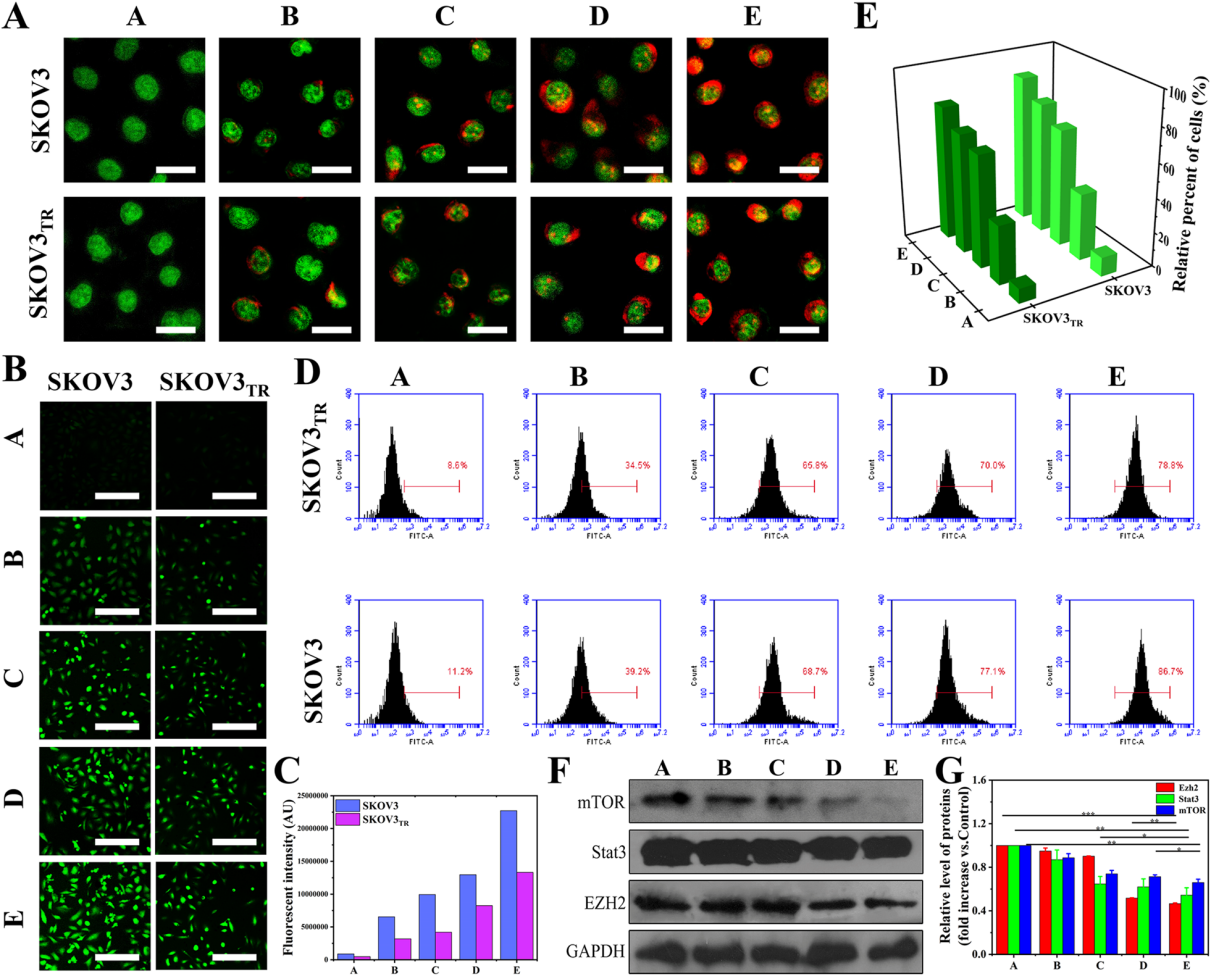
**A** Fluorescent images of AO-stained SKOV3 and SKOV3_TR_ cells treated with various therapeutic nanocomposites for 24 h. Scale bar = 50 μm. **B** Fluorescent images of Rhodamine 123-stained SKOV3 and SKOV3_TR_ cells treated for 24 h with various therapeutic nanocomposites, and **C** corresponding quantitative analysis. Scale bar = 500 μm. **D** Flow cytometry analysis of intracellular ROS levels of SKOV3 and SKOV3_TR_ cells treated for 24 h with various therapeutic nanocomposites **(E)** and corresponding quantitative analysis. **F** Western blotting of mTOR-related proteins in SKOV3 and SKOV3_TR_ cells after treatment for 24 h with various therapeutic nanocomposites and **G** corresponding quantitative analysis: A, control; B, HA-miR *let-7a*-pGNR @MSN; C, HA-PTX-pGNR@MSN; D, PTX/miR *let-7a*-pGNR@MSN; E, HA-PTX/miR *let-7a*-pGNR@MSN. AO, acridine orange; ROS, reactive oxygen species; mTOR, mammalian target of rapamycin; HA, hyaluronic acid; *let-7a*, *lethal-7a*; pGNR, polyethylene glycol-modified gold nanorod; MSN, mesoporous silica nanoparticle; PTX, paclitaxel

To determine the effects of various therapeutic nanocomposites on vesicles, SKOV3 and SKOV3_TR_ cells were stained with AO. Compared to the other groups, a significant increase in the number of induced red fluorescent spots in the cytoplasm was observed in the HA-PTX/miR *let-7a*-pGNR@MSN group, indicating a decrease in acidic compartments such as lysosomes and autophagic lysosomes (Fig. 6A).

Electrochemical potential energy is stored in the inner membrane of mitochondria. If there is an asymmetric distribution of proton plasma concentration on both sides of the membrane, the mitochondrial membrane potential shows an upward trend, which is called “MMP”. HA-PTX/miR *let-7a*-pGNR@MSN an increase in intracellular green fluorescent aggregates, suggesting depolarization of mitochondrial intima (Fig. 6B, C). The depolarization process of MMP will cause the mitochondria to release a large amount of cytochrome c into the cytoplasm, thus accelerating the process of apoptosis. In contrast, intracellular ROS levels were significantly elevated, consistent with earlier results (Fig. 6D, E).

Mammalian target of rapamycin (mTOR) is an important regulator of cell growth and proliferation [37–39]. Abnormal regulation of the mTOR signaling pathway is closely related to cell proliferation. Signal transducers and transcription activators (STAT) are a highly conserved family of transcription factors that are stimulated by extracellular signals for pure phosphorylation and translocation in the cytoplasm. STAT3 is one of the seven members of the STAT family and is involved in cell cycle, apoptosis regulation, tumor angiogenesis, tumor cell invasion, metastasis, and immune escape [40, 41]. Enhancer of zeste homolog 2 (EZH2), which is highly expressed in tumor-resistant cells and has histone methyltransferase activity, is involved in X chromosome inactivation, cell differentiation, and regulation of embryo development [42].

In this study, it was found that the decreased mTORC1 expression leads to a rapid decline in STAT3 level and a decrease in the protein phosphorylation it regulates, thereby reducing the histone methyltransferase activity of EZH2. These cascade reactions may be strong evidence that HA-PTX/miR let-7a-pGNR@MSN reverses MDR and inhibits the proliferation of SKOV3_TR_ cells (Fig. 6F, G).

### Reversal of MDR in ovarian cancer in vivo

**Fig. 7:**
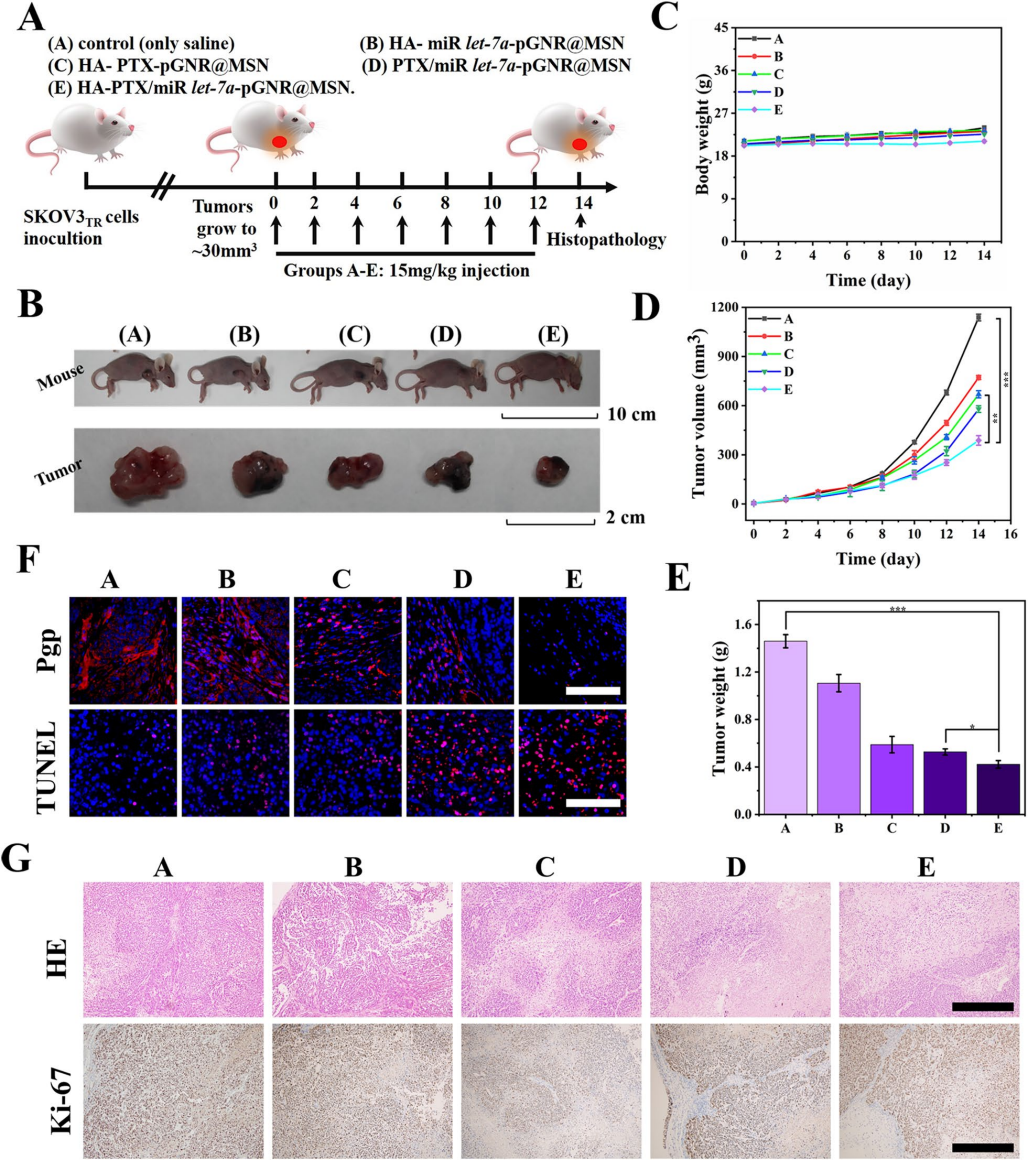
Therapeutic efficacy of various nanocomposites in vivo. **A** Experimental procedure designed for nanocomposite therapy. **B** Body weight curve of mice after intratumoral injection with various therapeutic nanocomposites following the experimental scheme. **C** Digital photographs of mice and dissected tumors. **D** Time-dependent tumor growth curves (*n* = 5, mean ± SD). **E** Tumor weight after 15 days of treatment. **F** P-gp immunohistochemical staining and TUNEL staining for analysis of pathological changes. Scale bar = 100 μm. **G** H&E staining and Ki-67 immunohistochemical staining for cellular proliferation analysis in tumor tissue after 15 days of treatment. Scale bar = 500 µm. NP, nanoparticle; SD, standard deviation; TUNEL, terminal deoxynucleotidyl transferase dUTP nick end labeling; P-gp, P-glycoprotein; H&E, hematoxylin and eosin

HA-PTX/miR let-7a-pGNR@MSN showed favorable ability of reversing MDR in vitro. Therefore, SPF SKOV3_TR_ BALB/c-nu mice were selected to establish a subcutaneous tumor model and treated with various therapeutic nanocomposites (15 mg/kg) every 2 days (Fig. 7A). After 15 days of treatment, the mice were euthanized and their main organs and tumor tissues were collected. As shown in Fig. 7B, there was no significant change in the body weight of all the mice, indicating that HA-pGNR@MSN nanocarriers had no significant toxicity to mice during the treatment. It was also shown that the HA-PTX/miR *let-7a*-pGNR@MSN group demonstrated better therapeutic effect compared to the PTX/miR *let-7a*-pGNR@MSN group. In addition, co-delivery of PTX/miR using HA-pGNR@MSN as nanocarriers effectively inhibited the growth of SKOV3_TR_ after intratumoral administration, which had a synergistic inhibitory effect on tumor growth (Fig. 7C–E).

**Fig. 8:**
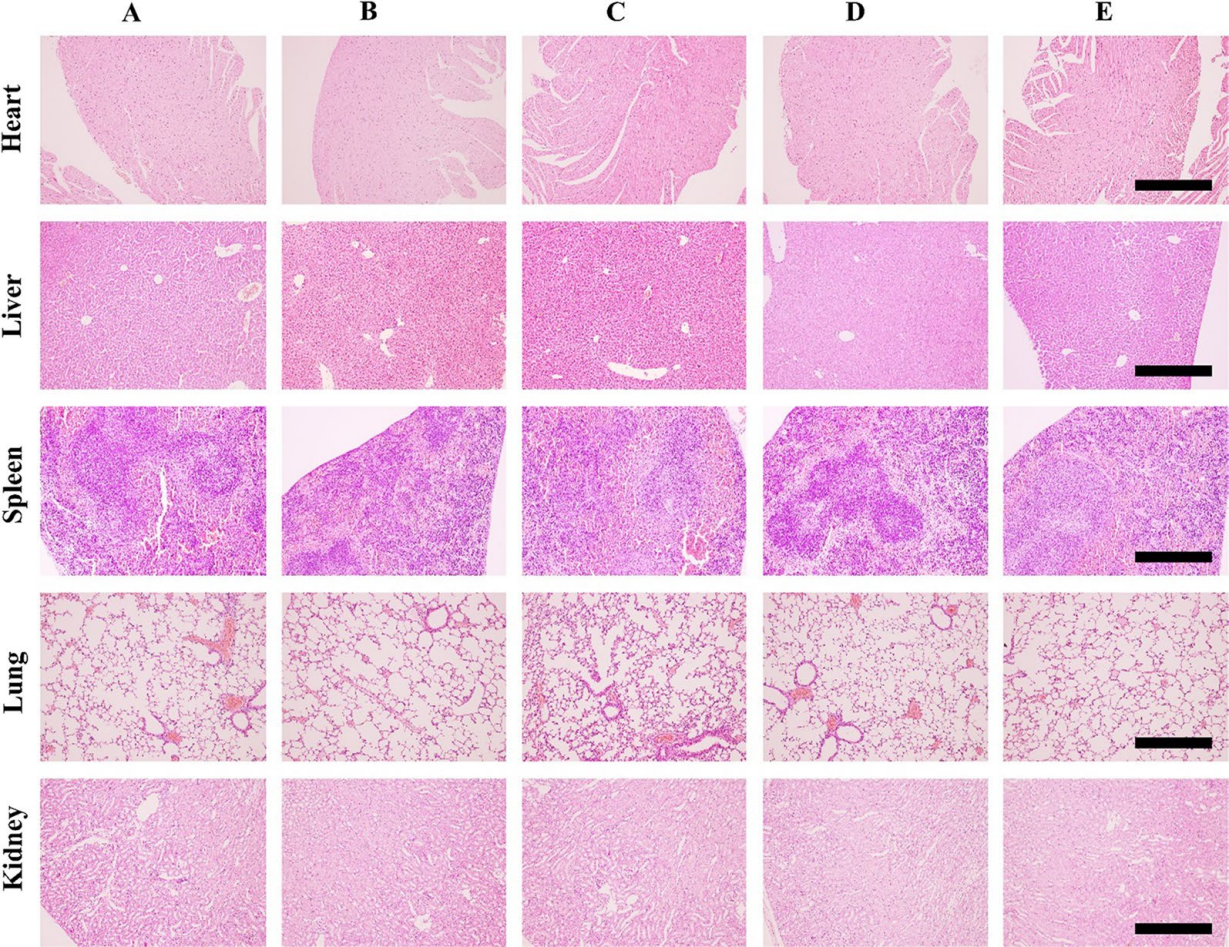
Representative images of H&E staining of main organs (heart, liver, spleen, lungs, and kidneys) of mice after 15 days of treatment. Scale bar = 500 µm. H&E, hematoxylin and eosin

The mechanism was further analyzed using antigen P-gp and TUNEL staining assay. After treatment with HA-PTX/miR *let-7a*-pGNR@MSN, the level of P-gp in the cancer tissue significantly decreased, which was consistent with in vitro results (Fig. 7F). TUNEL staining results showed that the staining signal decreased, indicating that the apoptosis of cancer cells of the HA-PTX/miR *let-7a*-pGNR@MSN group increased compared to other groups (Fig. 7F). H&E staining assay clearly showed significant destruction of solid tumors in the HA-PTX/miR *let-7a*-pGNR@MSN group (Fig. 7G). The decrease of Ki-67 level in the tumor tissue also indicated that the proliferation ability and malignancy of tumors decreased after treatment with HA-PTX/miR *let-7a*-pGNR@MSN (Fig. 7G). In addition, H&E staining showed the complete structure of major organs and no obvious tumor invasion and metastasis, indicating that HA-PTX/miR *let-7a*-pGNR@MSN had no obvious damage to major organs, further indicating that the nanoparticles had low toxicity and good safety (Fig. 8).

## Conclusions

In summary, we successfully developed a novel HA-modified targeted nanosystem pGNR@MSN for co-delivery of miR *let-7a* and PTX to overcome MDR in ovarian cancer and enhance chemotherapy effect. Our experimental results demonstrated that PTX and miR *let-7/*PTX could be effectively co-delivered to SKOV3/SKOV3_TR_ cells and ovarian cancer tissue by this nanosystem. Moreover, miR *let-7a* decreased P-gp expression, reversed the MDR of SKOV3_TR_ cells and ovarian cancer tissue, and enhanced the therapeutic effect of PTX, leading to high therapeutic efficiency. In the mantime, in vivo experiments showed that the delivery system had almost no toxicity. In-depth analysis of the experimental data implied that mTOR-mediated signaling pathways played an important role in reversing drug resistance and subsequent inducting apoptosis. Hence, this study shall provide theoretical guidance and effective basis for treatment of ovarian cancer in the future.

## Supplementary Information

**Additional file 1.** Additional Figures S1–S6.

## Data Availability

All data generated or analyzed during this study are included in this published article (and also in Additional information).

## Abbreviations

MDR: Multidrug resistance
P-gp: P-glycoprotein
miR: MicroRNA
*let-7a*: *lethal-7a*
PTX: Paclitaxel
HA: Hyaluronic acid
GNR: Gold nanorods
ATP: Adenosine triphosphate
MSN: Mesoporous silica nanoparticle
PEG: Polyethylene glycol
pGNR: Polyethylene glycol-modified gold nanorods
TEOS: Tetraethyl orthosilicate
FBS: Fetal bovine serum
RPMI: Roswell Park Memorial Institute
AO: Acridine orange
FDA: Fluorescein diacetate
PI: Propidium iodide
DAPI: 4ʹ,6-Diamidino-2-phenylindole
MTT: 3-(4,5-Dimethylthiazlo-2-diphenyl-tetrazolium) bromide
RBITC: Rhodamine B isothiocyanate
DMSO: Dimethyl sulfoxide
FITC: Fluorescein isothiocyanate
CTAB: Cetyltrimethylammonium bromide
DI: Deionized
APTES: 3-Aminopropyltriethoxysilane
HPLC: High-performance liquid chromatography
EDC: 1-Ethyl-3-(3-dimethylaminopropyl) carbodiimide hydrochloride
NHS: *N*-Hydroxysuccinimide
MES: 2-(*N*-Morpholino)ethanesulfonic acid
RT: Room temperature
HAADF-STEM: High-angle annular dark-field scanning transmission electron microscopy
HRTEM: High-resolution transmission electron microscopy
DLS: Dynamic laser scattering
FT-IR: Fourier transform infrared
XRD: X-ray diffraction
PBS: Phosphate-buffered saline
FAM: Carboxyfluorescein
MMP: Mitochondrial membrane potential
ROS: Reactive oxygen species
DCFH-DA: 2ʹ-7ʹDichlorofluorescin diacetate
SDS: Sodium dodecyl sulfate
PMSF: Phenylmethylsulfonyl fluoride
SDS-PAGE: Sodium dodecyl sulfate-polyacrylamide gel electrophoresis
PVDF: Polyvinylidene difluoride
PBST: Phosphate-buffered saline-Tween 20
HRP: Horseradish peroxidase
GADPH: Glyceraldehyde 3-phosphate dehydrogenase
SPF: Specific pathogen-free
H&E: Hematoxylin and eosin
TUNEL: Terminal deoxynucleotidyl transferase dUTP nick end labeling
EDS: Energy-dispersive X-ray spectroscopy
SSA: Specific surface area
RBC: Red blood cell
TEM: Transmission electron microscopy
Bcl-2: B-cell lymphoma 2
Mcl-1: Myeloid leukemia factor-1
Bax: Bcl-2-associated X protein
Bcl-XL: B-cell lymphoma extra large
PS: Phosphatidylserine
mTOR: Mammalian target of rapamycin
STAT: Signal transducer and activator of transcription
NP: Nanoparticle
SD: Standard deviation
