# Supplementary material for: A novel targeted co-delivery nanosystem for enhanced ovarian cancer treatment via multidrug resistance reversion and mTOR-mediated signaling pathway
Source: J Nanobiotechnology. 2021 Dec 23;19:444. doi: 10.1186/s12951-021-01139-1 (PMC8697442; doi:10.1186/s12951-021-01139-1)
Supplement: Supplementary file 1 — Additional file 1. Additional Figures S1–S6. [file 12951_2021_1139_MOESM1_ESM.docx]

**Supporting Information**

**A novel targeted co-delivery nanosystem for enhanced ovarian cancer treatment via multidrug resistance reversion and mTOR-mediated signaling pathway**

Xueqin Wang^1, 2^, Tiandi Xiong^2^, Miao Cui^2^, Na Li^1, 3^, Qin Li^1, 3^, Li Zhu^1, 3^, Shaofeng Duan^5, 6^ *,

Yunlong Wang^7^*and Yuqi Guo^1,3,4^ *

^1^*Henan Provincial People's Hospital, Zhengzhou, 450003, China.* ^2^*College of Bioengineering, Henan University of Technology, Zhengzhou, 450001, China.* ^3^*People's Hospital of Zhengzhou University, Zhengzhou, 450003, China.* ^4^*Henan International Joint Laboratory for Gynecological Oncology and Nanomedicine, Zhengzhou, 450003, China.* ^5^*Institute for innovative drug design and evaluation, School of Pharmacy, Henan University, Kaifeng, 475004, China.* ^6^*Henan International Joint Laboratory of Chinese medicine efficacy, Henan University, Kaifeng, 475004, China.* ^7^*Henan Bioengineering Research Center, Zhengzhou, 450046, China.*

* *Corresponding authors: Shaofeng Duan, E-mail address*: [*sduan@henu.edu.cn*](mailto:sduan@henu.edu.cn)*; Yunlong Wang, E-mail address*: [*biowyl@126.com*](mailto:biowyl@126.com); *Yuqi Guo, E-mail address*: *[yuqi-guo@163.com](mailto:yuqi-guo@163.com), Tel.: + 86 371 65580059; fax: + 86 371 65580059*

**Materials**

The duplexed unlabeled miR *let-7a*, 50-carboxyfluorescein (FAM) labeled and scrambled miR *let-7a* were synthesized with the following sequences by Shanghai Gene Pharma Co., Ltd (sense, 5’-UGAGGUAGUAGGUUGUAUAGUU-3’; mimmics, sense, 5’- UGAGGUAGUAGGUUGUAUAGUU-3’; inhibitors, sense, 5’- AACUAUACAACCUACUACCUCA -3’).

**Stability of HA-pGNR @ MSN nanosystem in different solutions**

The stability of HA-pGNR @ MSN nanosystem suspended in various solutions including RMPI-1640 with 10% FBS, PBS (pH 7.4), DI water, was estimated at different settling times. We suspended 20 mL of HA-pGNR @ MSN (10 mg/mL) in RMPI-1640 with 10% FBS, PBS (pH 7.4), DI water, respectively. Subsequently, absorbance of the supernatants was determined employing an UV-visible spectrophotometer (UV-1000, Shanghai, China) at 480 nm.

**The drug PTX release**

The release behavior of PTX from HA-PTX/*let-7a*-GNR@MSN was evaluated using a dialysis method [1,2]. The releasing of PTX from the HA-PTX/*let-7a*-GNR@MSN was evaluated in the PBS solution with representative pH including (pH 5.0 and 7.4). In brief, the HA-PTX/*let-7a*-GNR@MSN (final concentration: 1 mg mL^-1^) and free PTX (0.05 mM) were placed in a dialysis bag (MWCO: 20 kDa) and dispersed in 30 mL PBS, and incubated at different pHs (5.0 or 7.4) with gentle stirring at 37 ℃ for 72 h, followed with dialysis. At predetermined time points, 2 mL of dialysates was taken out and replaced with same volume of fresh liquid. The dynamic change of drug residual on the HA-PTX/*let-7a*-GNR@MSN was measured at 235 nm with an UV-visible spectrophotometer (UV-1000, Shanghai, China).


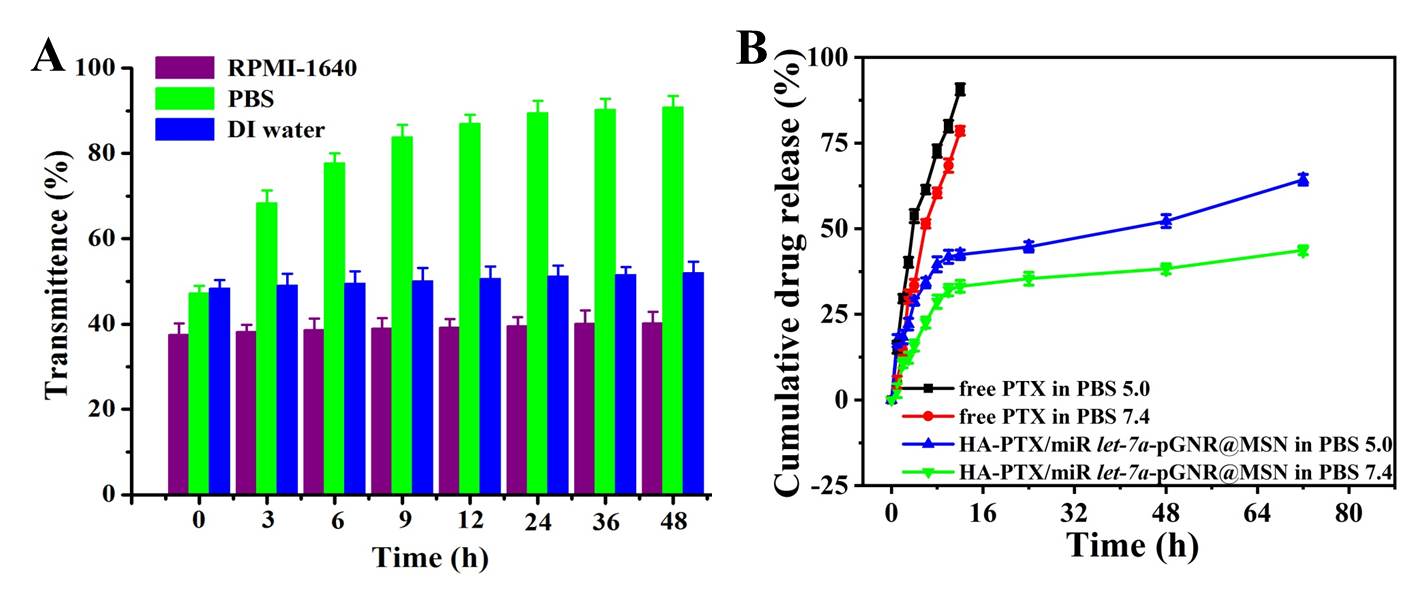


**Fig. S1** (A) Stability assay of the HA-pGNR @ MSN nano co-deliverysystem in aqueous solution including 1640 medium, PBS (pH 7.4), and DI water. (B) Release of PTX from HA-PTX/ miR *let-7a*-GNR@MSN was evaluated in the PBS solution with representative pH including (pH 5.0 and 7.4).


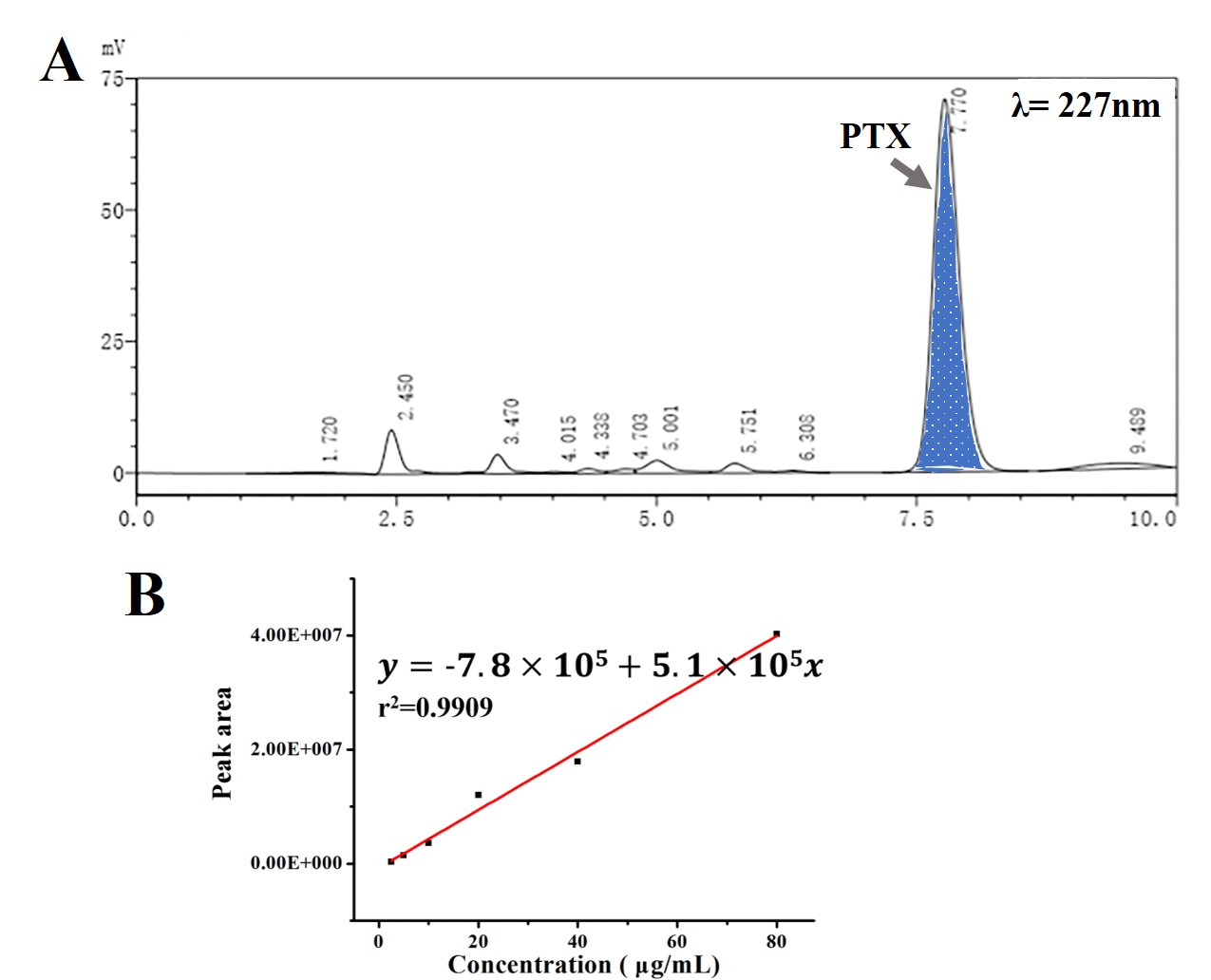


**Fig. S2 (A)** Peak plot of PTX in HPLC. **(B)** Standard curve of PTX content.


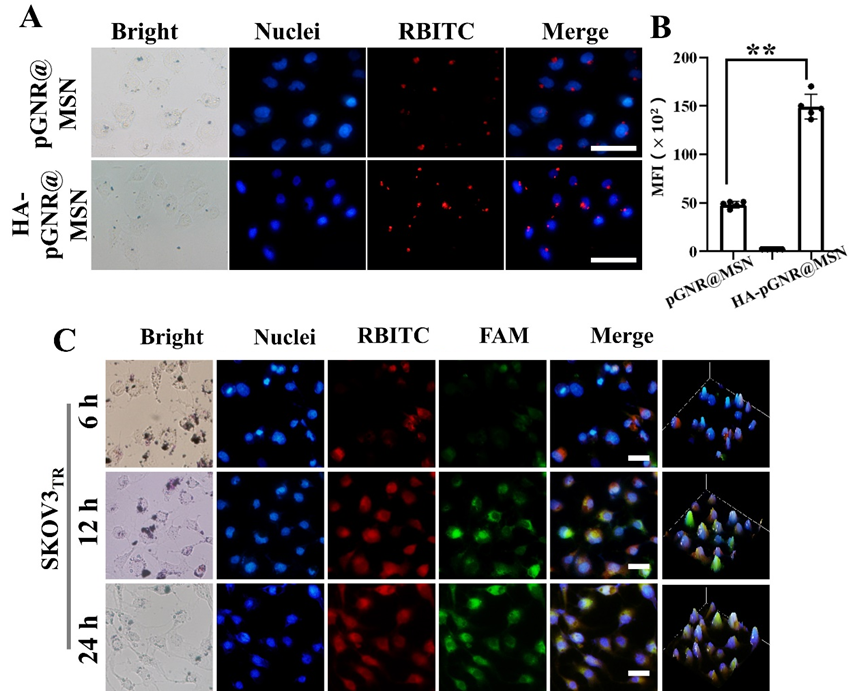


**Fig. S3** Cellular uptake assay. **(A)** Fluorescent images of ^RBITC^pGNR@MSN and ^RBITC^HA- pGNR @MSN after 12 h treatment of SKOV3 cells (concentration 50 μg/mL) and **(B)** corresponding data analysis. **(C)** Fluorescent images of HA-^FAM^ miR *let-7a*-^RBITC^pGNR@MSN after for 6, 12, and 24 h treatment of SKOV3_TR_ cells: FAM (green), RBITC (red), and DAPI (blue). Scale bar = 50 μm. pGNR, polyethylene glycol–modified gold nanorod; MSN, mesoporous silica nanoparticle; HA, hyaluronic acid; *let-7a*, *lethal-7a*; FAM, carboxyfluorescein; RBITC, Rhodamine B isothiocyanate; and DAPI, 4',6-diamidino-2-phenylindole.

*
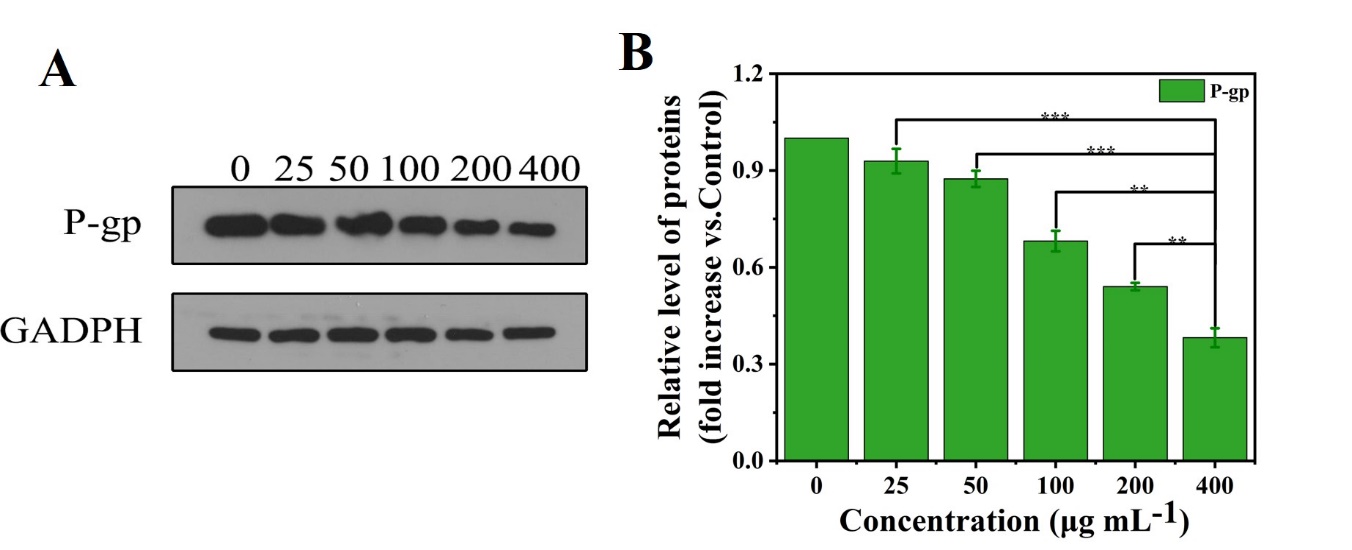
*

**Fig. S4 (A)** Western blotting assay was used to measure the P-gp expression after treatment for 24 h with different concentrations of nanocomposites including 0, 25, 50, 100, 200 and 400 μg/mL, and **(B)** corresponding quantitative analysis.


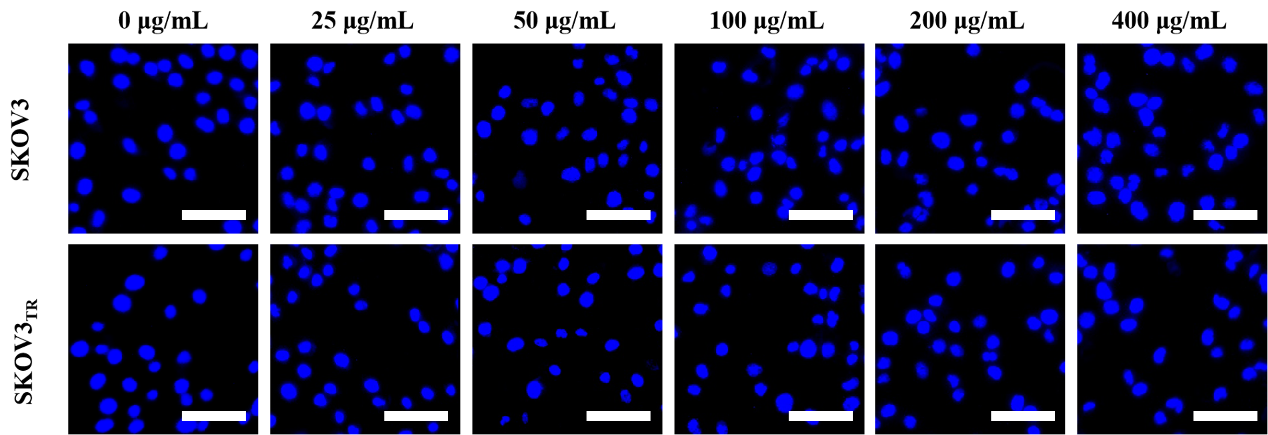


**Fig. S5** Hoechst H33258 staining fluorescence images of the treated SKOV3 and SKOV3_TR_ using different concentration HA-PTX/miR *let-7a*-pGNR@MSN. Scale bar = 100 μm.

*
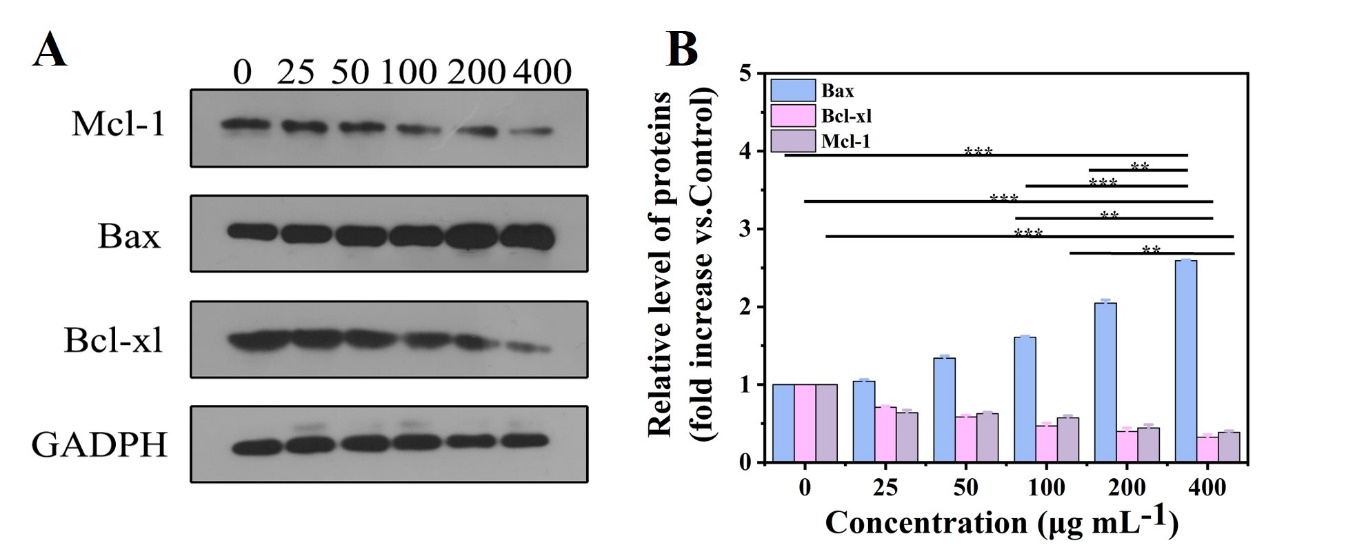
*

**Fig. S6 (A)** Western blot analysis of apoptosis-associated proteins in the treated SKOV3_TR_ cells with various concentrations of HA-PTX/miR *let-7a*-pGNR@MSN (0, 25, 50, 100, 200 and 400 μg/mL), and **(B)** statistical analysis of relative level of proteins.

**Reference**

1. Vajedi FS, Dehghani H, Zarrabi A. Design and characterization of a novel pH-sensitive biocompatible and multifunctional nanocarrier for in vitro paclitaxel release. Mater Sci Eng C 2021; 119:111627.

2. Katiyar SS, Ghadi R, Kushwah V, Dora C P, Jain S. Lipid and biosurfactant based core-shell-type nanocapsules having high drug loading of paclitaxel for improved breast cancer therapy. ACS Biomater Sci Eng 2020; 6: 6760-9.
